# Supplementary material for: A ketogenic diet sensitizes pancreatic cancer to glutamine metabolism inhibitors
Source: Cell Rep Med. 2026 Apr 23;7(5):102770. doi: 10.1016/j.xcrm.2026.102770 (PMC13198290; doi:10.1016/j.xcrm.2026.102770)
Supplement: Document S1. Figures S1–S5 [file mmc1.pdf]

Cell Reports Medicine, Volume 7

Supplemental information

A ketogenic diet sensitizes pancreatic cancer to glutamine metabolism inhibitors

Omid Hajihassani, Asael Roichman, Jacob A. Boyer, Michal MacArthur, Ricardo Cordova, Alexander Loftus, Christina S. Boutros, Jonathan J. Hue, Parnian Naji, Soubhi Tahhan, Peter Gallagher, William Beegan, Danyal Shah, James Choi, Nimat Manzoor, Shihong Lei, Christine Kim, Moeez Rathore, Ishan Shah, Kevin Lebo, Helen Cheng, Anusha Mudigonda, Craig Hunter, Mehrdad Zarei, Sydney Alibeckoff, Karen Ji, Hallie Graor, Masaru Miyagi, Ali Vaziri-Gohar, Henri Brunengraber, Rui Wang, Peder J. Lund, Luke D. Rothermel, Joshua D. Rabinowitz, and Jordan M. Winter

Supplemental figure titles and legends:

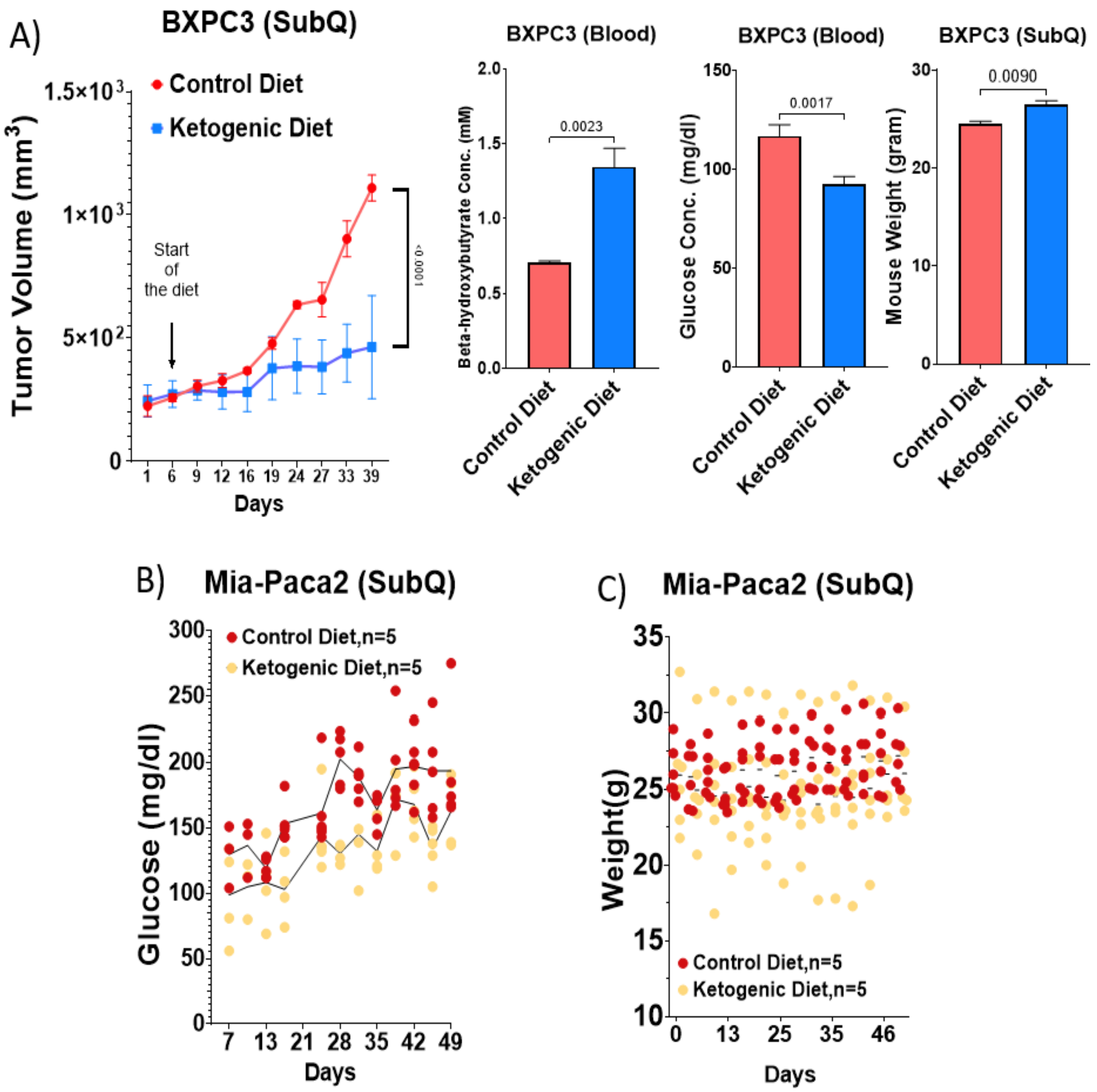

Supplementary Fig. 1: Mice maintained on a ketogenic diet reveal lower blood glucose levels while effectively managing their body weight. Related to figure 1. **(A)** BXPC3 tumors injected in the flank of athymic nude mice. Glucose levels were measured in individual mice throughout the experiment. During the experiment the levels of BHB, glucose and mouse weight were collected (n=5). **(B)** Tumor volumes per mouse are illustrated throughout the duration of the experiment, with each day's measurement representing n=5. **(C)** Mouse body weights were measured twice per week (n=5).

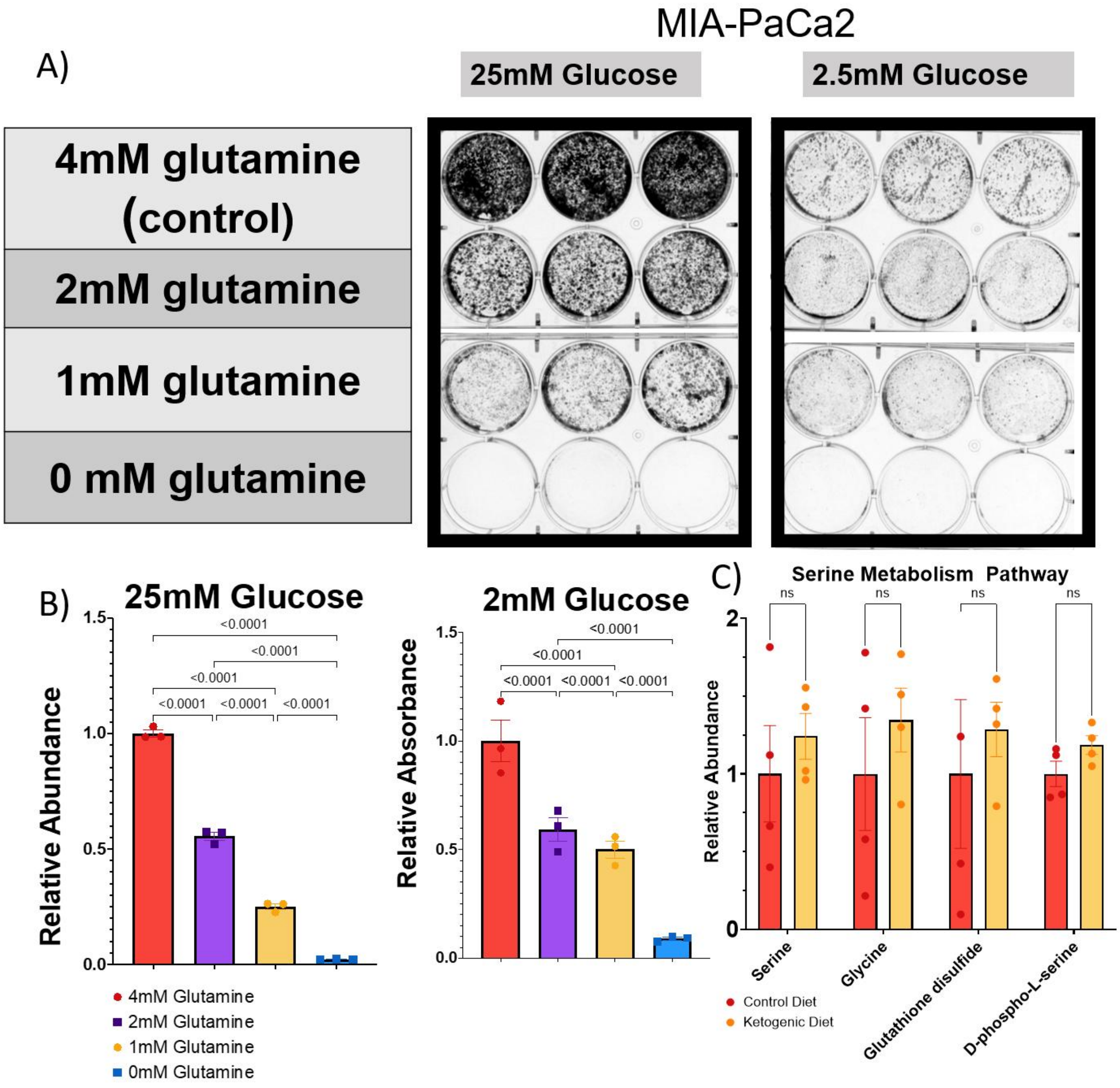

Supplementary Fig. 2: Glutamine is required for pancreatic cancer cells viability. Related to figure 2. Different glutamine levels were tested under high (25mM) and low (2.5mM) glucose conditions (n=3). **(B)** Quantitation of cell viability at the different indicated conditions (n=3). **(C)** Serine metabolism (n=3).

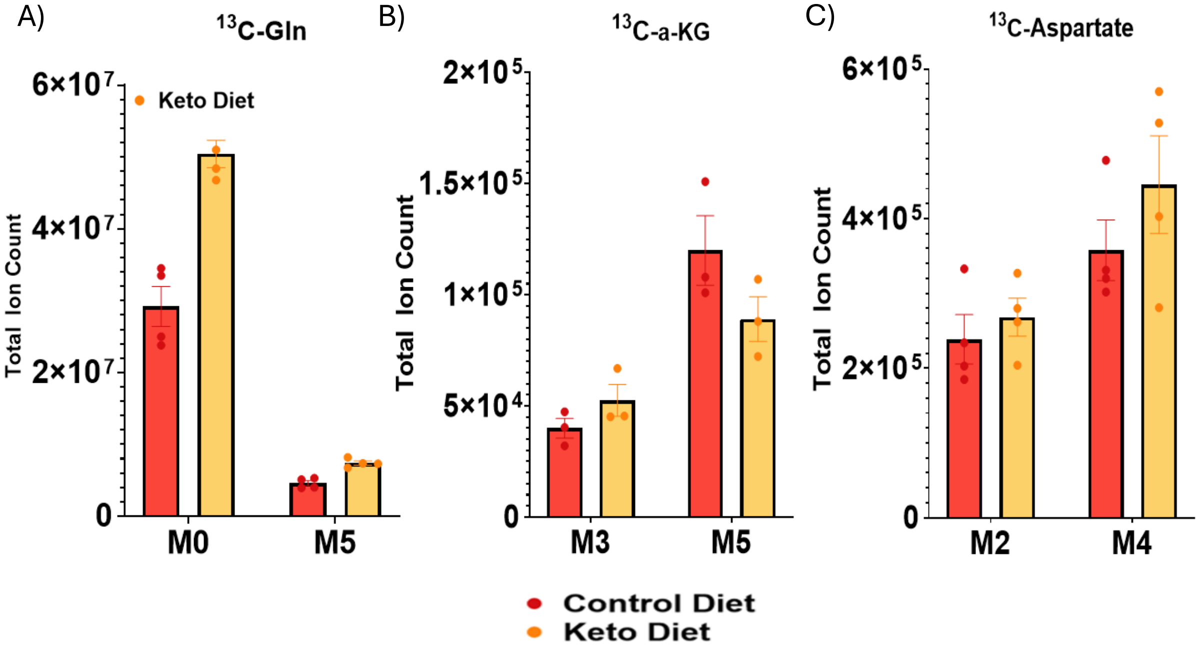

Supplementary Figure 3. PDAC tumors exhibit increase glutamine utilization under a ketogenic diet *in vivo.* Related to figure 4. Nude mice bearing PDAC flank tumors were injected with uniformly labeled [^{13}C]-glutamine and analyzed after 2.5 hr of tracer circulation(n=3). **(A)** M+0 and M+5 isotopologues of glutamine, and relevant isotopologues (n=3) of **(B)** α-ketoglutarate (n=3), and **(C)** aspartate (n=3).

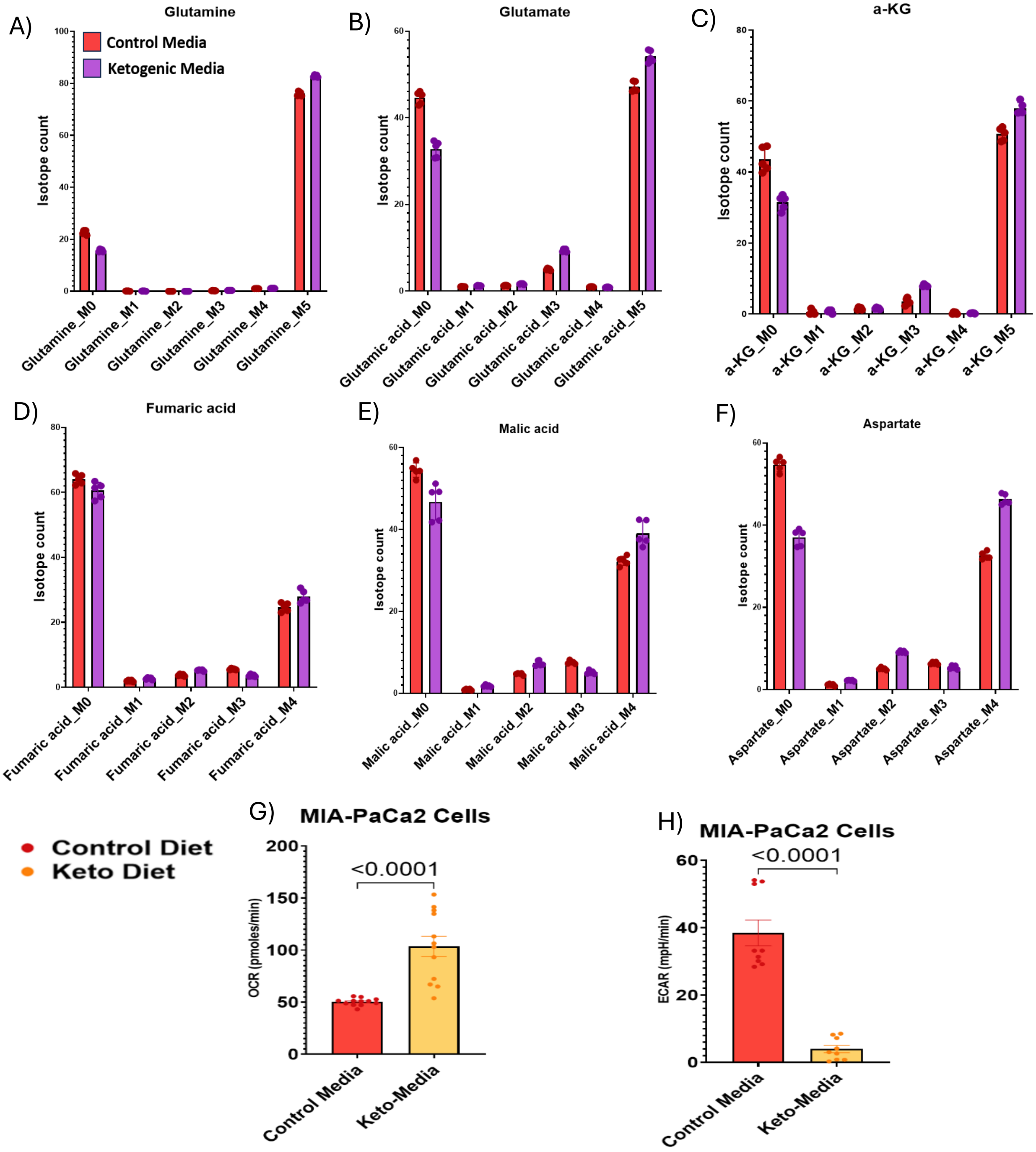

Supplementary Figure 4. PDAC cells maintain higher levels of TCA cycle metabolites under ketogenic conditions *in vitro.* Related to figure 5. Isotopologue analysis of [^{13}C]-glutamine metabolism was performed using 6 mM glutamine in ketogenic media and 4 mM glutamine in control media (n=3). **A)** M+0 through M+5 isotopologues of glutamine under control and ketogenic conditions (n=3). **B)** Glutamate, **C)** α-ketoglutarate, and M+0 through M+4 D**)** fumarate, **E)** malate, and **F)** aspartate. Seahorse quantified levels of **(G)** OCR **(H)** ECAR for cells treated with ketogenic media vs. cells treated with control media.

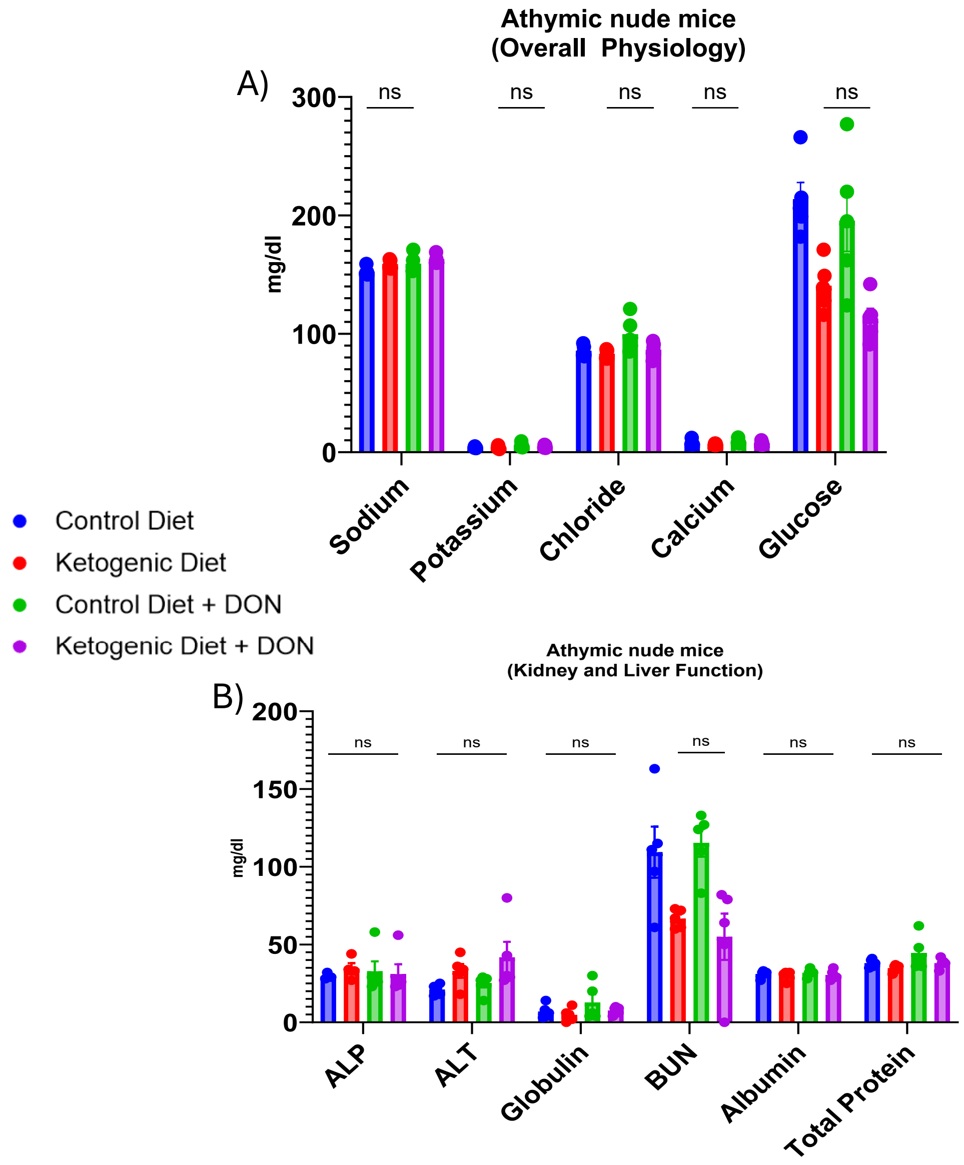

Supplementary Fig.5: Ketogenic diet and DON combination therapy reveals no significant toxicity. Related to figure 6. Tumor-bearing mice were evaluated (n=5). **(A)** Electrolyte and glucose levels reveal no significant differences in treatment groups. **(B)** Alkaline Phosphatase (ALP), Alanine aminotransferase (ALT), globulin, blood urea nitrogen (BUN), albumin, and total protein were measured as surrogate markers of liver and kidney function (n=5).
