# Supplementary material for: A ketogenic diet sensitizes pancreatic cancer to glutamine metabolism inhibitors
Source: Cell Rep Med. 2026 Apr 23;7(5):102770. doi: 10.1016/j.xcrm.2026.102770 (PMC13198290; doi:10.1016/j.xcrm.2026.102770)
Supplement: Document S2. Article plus supplemental information [file mmc2.pdf]

Cell Reports Medicine

Article

A ketogenic diet sensitizes pancreatic cancer to glutamine metabolism inhibitors

Graphical abstract

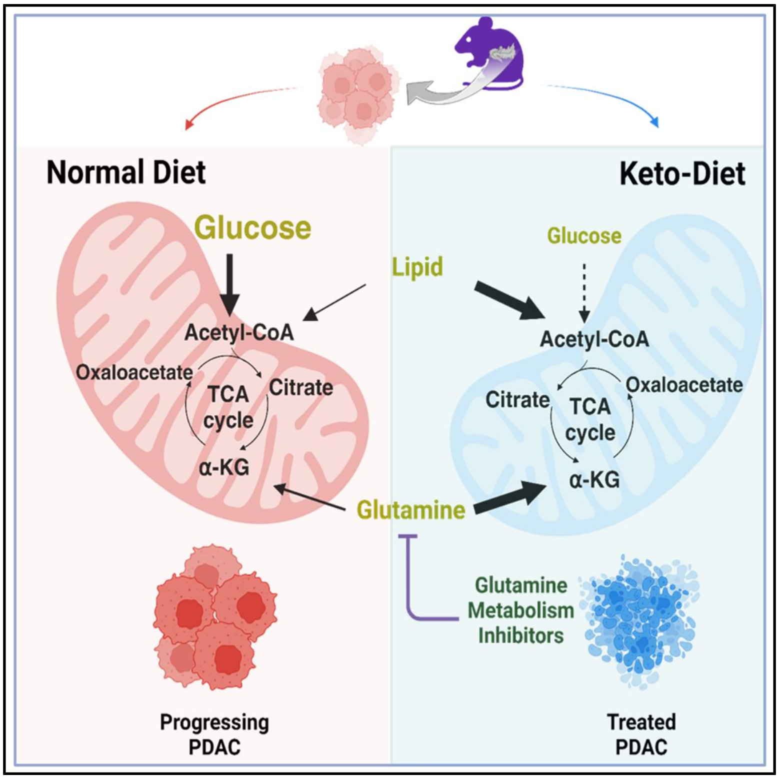

Highlights

- Ketogenic diet rewires PDAC metabolism by altering nutrient availability
- Ketogenic diet increases PDAC dependence on glutamine, fatty acids, and ketone bodies
- Glutamine inhibition synergizes with ketogenic diet to suppress tumors without toxicity

Authors

Omid Hajihassani, Asael Roichman, Jacob A. Boyer, ..., Luke D. Rothermel, Joshua D. Rabinowitz, Jordan M. Winter

Correspondence

jordan.winter@uhhospitals.org

In brief

Hajihassani et al. demonstrate that a ketogenic diet reprograms PDAC metabolism by altering nutrient availability, increasing reliance on glutamine, fatty acids, and ketone bodies to sustain core metabolic pathways. Combining a ketogenic diet with glutamine utilization enhances anti-tumor efficacy without inducing significant toxicity.

Hajihassani et al., 2026, Cell Reports Medicine *7*, 102770
May 19, 2026 © 2026 The Author(s). Published by Elsevier Inc.
https://doi.org/10.1016/j.xcrm.2026.102770

CellPress

Cell Reports Medicine

CellPress
OPEN ACCESS

Article

A ketogenic diet sensitizes pancreatic cancer to glutamine metabolism inhibitors

Omid Hajihassani,[1,2] Asael Roichman,[3,4,10] Jacob A. Boyer,[3,6] Michal MacArthur,[3,4] Ricardo Cordova,[4,6] Alexander Loftus,[2] Christina S. Boutros,[2] Jonathan J. Hue,[2] Parnian Naji,[1,2] Soubhi Tahhan,[2] Peter Gallagher,[2] William Beegan,[2] Danyal Shah,[2] James Choi,[1] Nimat Manzoor,[1] Shihong Lei,[1] Christine Kim,[1] Moeez Rathore,[2] Ishan Shah,[1] Kevin Lebo,[1] Helen Cheng,[1] Anusha Mudigonda,[1] Craig Hunter,[3,4,5] Mehrdad Zarei,[1] Sydney Alibeckoff,[9] Karen Ji,[1] Hallie Graor,[1,2] Masaru Miyagi,[1] Ali Vaziri-Gohar,[8] Henri Brunengraber,[1] Rui Wang,[1,2] Peder J. Lund,[1,2,7] Luke D. Rothermel,[2] Joshua D. Rabinowitz,[3,4,5,6] and Jordan M. Winter[1,2,11,*]

[1]Case Comprehensive Cancer Center, Case Western Reserve University, Cleveland, OH, USA
[2]Department of Surgery, Division of Surgical Oncology, University Hospitals, Cleveland Medical Center, Cleveland, OH, USA
[3]Princeton University, Princeton, NJ, USA
[4]Department of Chemistry, Princeton University, Princeton, NJ 08544, USA
[5]Lewis-Sigler Institute of Integrative Genomics, Princeton University, Princeton, NJ, USA
[6]Ludwig Institute for Cancer Research, Princeton Branch, Princeton, NJ, USA
[7]Department of Nutrition, Case Western Reserve University, Cleveland, OH, USA
[8]Departments of Cancer Biology and Surgery, Stritch School of Medicine, Loyola University Chicago, Chicago, IL 60153, USA
[9]Department of Molecular and Integrative Physiology, University of Michigan School of Medicine, Ann Arbor, MI, USA
[10]The Mina and Everard Goodman Faculty of Life Sciences, Bar-Ilan University, Ramat-Gan, Israel
[11]Lead contact
*Correspondence: jordan.winter@uhhospitals.org
https://doi.org/10.1016/j.xcrm.2026.102770

SUMMARY

Pancreatic cancer is the third leading cause of cancer-related death in the United States. Current chemotherapy options provide limited benefits. Emerging evidence suggests that a ketogenic diet (KD) exerts anti-tumor effects by reprogramming tumor metabolism and revealing therapeutic vulnerabilities. Efforts to target glutamine metabolism—an essential pathway in many cancers—have shown promise in preclinical models, but clinical efficacy has remained limited. Here, we show that a KD increases tricarboxylic acid (TCA) cycle activity and elevates reliance on glutamine-related metabolites in murine pancreatic cancer models and *in vitro* under KD-mimicking conditions. This metabolic adaptation occurs in response to reduced glucose availability. We demonstrate that combining glutamine metabolism inhibitors, such as CB-839 or 6-diazo-5-oxo-L-norleucine (DON), with a KD leads to robust anti-tumor effects in preclinical models of pancreatic cancer. Thus, metabolic vulnerability induced by dietary intervention provides a rationale for combining glutamine-targeted therapies with a ketogenic diet in future clinical studies.

INTRODUCTION

Pancreatic ductal adenocarcinoma (PDAC) has a poor prognosis, with a 5-year overall survival rate of approximately 12%.[1] A complex and dense tumor microenvironment (TME) is a prominent contributor to the aggressive biology of PDAC.[2–4] For instance, glutamine and glucose levels are depressed at baseline in the PDAC TME.[5] A haphazard microvasculature and associated steep nutrient gradients activate adaptive metabolic reprogramming and survival pathways in PDAC cells, which drive treatment resistance and promote cancer progression.[6] Numerous studies have shown that PDAC cells adapt to a challenging and nutrient-limited TME by reprogramming metabolism toward more efficient energy extraction. This metabolic shift involves a greater utilization of oxidative phosphorylation by mitochondria to maximally extract ATP for pro-survival pathways when nutrients are scarce.[7–9]

A ketogenic diet, alone or in combination with other therapies, has displayed an anti-cancer signal across a wide array of cancer subtypes in clinical and pre-clinical studies.[8,10] While dietary modification alone probably will not impact PDAC survival in a clinically meaningful way, the physiological changes induced by a ketogenic diet, particularly within a PDAC TME that is already nutrient-limited, may push conditions to a point that creates more exaggerated metabolic dependencies. These metabolic rewiring effects likely expose actionable therapeutic vulnerabilities in cancer cells that can be further exploited and that are absent in normal tissues. Thus, insights into the metabolic changes induced by a ketogenic diet could nominate specific therapeutic partners to potentiate anti-tumor effects of a ketogenic diet.[9–12] For instance, a ketogenic diet has been shown to increase circulating glutamine (Gln) and related metabolites in animal models, even though protein content in a ketogenic diet is low.[13,14] Endogenous glutamine production may be

© 2026 The Author(s). Published by Elsevier Inc.
This is an open access article under the CC BY license (http://creativecommons.org/licenses/by/4.0/).

upregulated due to lower levels of glutamine present in the diet.[15] Glutamine is widely recognized as a critical nutrient and plays a multifaceted role in cellular metabolism. For instance, the organic backbone of glutamine contributes to the generation of TCA cycle intermediates through anaplerosis via α-ketoglutarate,[16–18] and the amide nitrogen fuels the biosynthesis of essential metabolites like asparagine and nucleotides.[19,20]

Previous work by our group revealed that PDAC cells preferentially metabolize Gln when glucose levels are reduced.[21] Since one of the key impacts of a ketogenic diet is to lower systemic and intratumoral glucose levels due to substantially reduced carbohydrate intake,[11] we reasoned that this feature of a ketogenic diet might enhance reliance on glutamine metabolism as an alternative prioritized fuel source in PDAC cells.[22,23] Glutaminase (GLS) (which converts glutamine to glutamic acid prior to entry into the TCA cycle) inhibition, has been explored in pancreatic cancer models as a target of single-agent therapy, and the treatment transiently reduces cellular proliferation *in vitro*. However, this approach was ineffective across multiple *in vivo* PDAC models as a monotherapy due to compensatory metabolic reprogramming.[19] It was postulated that broadening the inhibition of Gln metabolism to related targets beyond GLS could enhance efficacy and thwart adaptive rewiring changes responsible for resistance. For instance, a Gln analog, 6-diazo-5-oxo-1-norleucine (DON), which covalently and irreversibly binds to multiple Gln-metabolizing enzymes, broadly impedes Gln metabolism to a superior degree, including its utilization in the generation of hexosamines and nucleotides.[16] These studies hint at the superiority of a less selective pharmacologic approach.

Herein, we explore whether a ketogenic diet exposes a vulnerability to glutamine metabolism inhibition through induction of a low glucose state in PDAC. Additionally, we attempt to illuminate how lipids and ketone bodies supplied by a ketogenic diet are utilized by cancer cells to compensate for low glucose and low protein levels, which could expose additional metabolic pathway targets beyond glutamine metabolism. This line of investigation offers an example of a "push-pulse" strategy, where a ketogenic diet nudges the system toward greater dependency on a specific metabolic program, thereby exposing new dependencies.[24] Our findings indicate that a ketogenic diet elevates circulating glutamine levels in both serum and tumor tissue. Tumors from mice fed a ketogenic diet exhibit increased glutamine uptake, driven by enhanced glutamine production. In response, tumors upregulate glutamine transporters and key genes involved in glutamine metabolism to support their metabolic needs. Combining a ketogenic diet with glutamine inhibition produced a potent anti-tumor effect *in vivo*. These results reveal a diet-induced metabolic vulnerability and provide a rationale for future clinical trials evaluating this combination strategy in pancreatic cancer.

RESULTS

A ketogenic diet inhibits tumor progression in mice

We tested the effects of a ketogenic diet on tumor growth and compared the findings to a control diet in a series of experiments involving MIA-PaCa2 (derived from human PDAC) xenografts in athymic nude mice or orthotopic pancreatic injection of KPC (derived from murine PDAC) xenografts in C57BL/6 mice. The control diet contained 7% calories from fat, while the ketogenic diet comprised 90% of calories from fat. We observed a robust suppression of PDAC growth associated with the ketogenic diet alone (i.e., no other treatments) across both models (Figures 1A and 1B; Figure S1A). The anti-tumor effect of the ketogenic diet in these experiments was stronger than that reported in many previous studies.[25,26] Mice on the ketogenic diet exhibited reductions in circulating glucose levels, maintaining a range of 100–160 mg/dL, compared to mice on the standard diet where glucose levels ranged from 140 to 220 mg/dL (Figure 1C; Figures S1A and S1B). We confirmed ketosis through measurements of circulating β-hydroxybutyrate levels (mean of 1.5 mM vs. mean of 0.5 mM in controls; Figure 1C). While certain published studies by others have shown a sharp drop in body weight when transitioning to a ketogenic diet,[27] we observed an initial drop of 10%–15% with a ketogenic diet, followed by weight stability beyond day 15 of the study (Figure 1D; Figure S1C).

A ketogenic diet enhances levels of glutamine and TCA cycle metabolites in serum and tumors

A ketogenic diet reprograms the metabolic landscape of PDAC tumors, and changes are detectable in both the tumors and sera of mice. Specifically, a ketogenic diet resulted in elevated intratumoral glutamine levels in MIA-PaCa2 tumors (Figure 2A). Additionally, intratumoral levels of glutamate (derived from glutamine by GLS), aspartate (generated from glutamate by GOT), ketone bodies, and TCA cycle metabolites (potentially fueled by glutamine-glutamate anaplerosis, ketone bodies, and fatty acids) were all increased (Figures 2A and 2B). Furthermore, increased dietary fat intake with a ketogenic diet translated to elevated levels of circulating fatty acids (Figure 2C). Changes in circulating glutamine levels in blood mirrored the increase in intratumoral glutamine (Figure 2D). Similarly, circulating ketone bodies in the serum correlated with increased intratumoral β-hydroxybutyric acid concentrations (Figure 2E). Not all fatty acids were elevated in the bloodstream (Figure 2F), while a select subset was increased in both sera and tumor tissue, such as C16:2, C18:1, and C18:2 fatty acids (Figure 2C). Collectively, these findings suggest a direct impact of a ketogenic diet on PDAC tumor glutamine metabolism, potentially fueling key metabolic functions like oxidative phosphorylation, despite diminished circulating glucose levels.

We next assessed the impact of glutamine availability on MIA-PaCa2 cells *in vitro*. The results demonstrated that a 50%-reduction in glutamine levels led to a corresponding 50% decrease in cell viability. This was even true under high glucose conditions (25 mM) characteristic of standard cell culture media (Figure S2A). Under low glucose conditions (2.5 mM, as a simulation of a key effect of a ketogenic diet on intratumoral glucose levels),[5] cell viability was markedly reduced, especially after a modest reduction in glutamine (Figure S2B). We observed minimal changes in metabolic pathways that originate from glycolytic intermediates, like serine metabolism (Figures 2C and S2C). These data indicate that a ketogenic diet profoundly impacts circulating and tumoral metabolite levels, and elevated glutamine could be an important metabolic adaptation to limited glucose availability.

CellPress
OPEN ACCESS

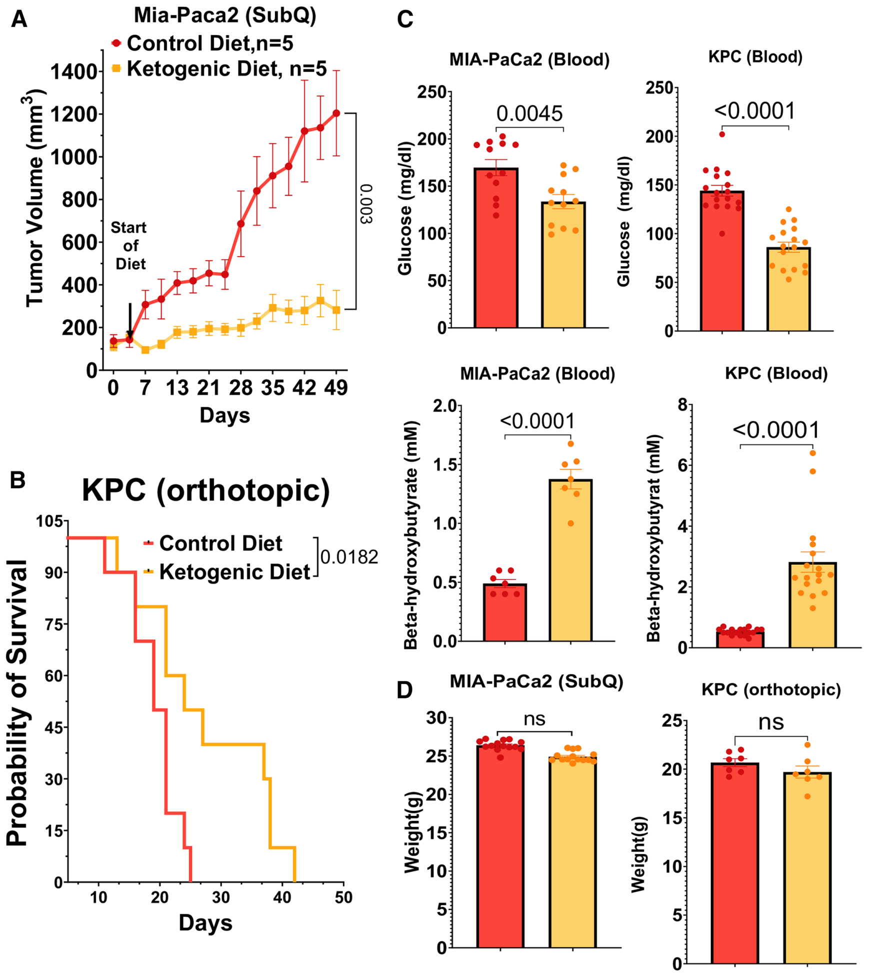

Figure 1. Suppression of tumor growth in PDAC models with a ketogenic diet

(A) Subcutaneous injection of MIA-PaCa2 cells in athymic nude mice ($n = 5$).

(B) Survival probability after orthotopic injection of KPC cells into the pancreas of C57BL/6 mice ($n = 10$).

(C) Levels of glucose and of β-hydroxybutyric acid in blood of C57BL/6 and athymic nude mice undergoing a normal diet and a ketogenic diet ($n = 5$).

(D) Body weights of treated mice compared to the control diet group ($n = 5$).

A low-protein ketogenic diet upregulates glutamine production and glutamine uptake in tumors

Under conditions of dietary protein restriction, the liver activates compensatory metabolic pathways to maintain systemic nitrogen balance and amino acid homeostasis.[28] Specifically, hepatic glutamine production is significantly upregulated through the glutamine synthetase (GS) pathway, serving as a critical adaptive mechanism to offset reduced dietary protein intake.[15] This physiological response becomes particularly relevant in the context of a ketogenic diet, which typically contains substantially lower protein levels (approximately 10%–15% of total calories) compared to the standard Western diet (20%–25% protein by calories).

Our investigations into the metabolic effects of this dietary intervention in PDAC-bearing mice revealed that MIA-PaCa2 pancreatic tumors exhibited metabolic plasticity in the context of a ketogenic diet. Tumor cells responded by upregulating the expression of ASCT2 (SLC1A5), the primary glutamine transporter (Figures 3A and 3B). This was accompanied by the coordinated upregulation of multiple genes involved in glutamine metabolism, including glutaminase (GLS1/2) and glutamic-oxaloacetic transaminase (GOT1), indicating broad reprogramming of glutamine utilization pathways (Figure 3C). These tumor-associated adaptations occurred in parallel with a significant elevation in hepatic glutamine synthetase expression, suggesting an increase in systemic glutamine synthesis (Figure 3D). As shown above, mass spectrometry analysis of blood samples confirmed a 3.5-fold increase in circulating glutamine levels in ketogenic-diet-fed mice compared to controls ($p < 0.01$) (Figure 2D). This finding suggests the activation of a liver-tumor metabolic axis, where the liver's compensatory glutamine production in response to a ketogenic diet meets the tumor's heightened glutamine demands under protein-restricted dietary conditions.

Glutamine uptake is increased in PDAC in mice and cell culture

We performed isotope-tracing studies to further characterize tumoral reliance on glutamine in the context of a ketogenic diet. Mice maintained on either a ketogenic or a control diet were administered uniformly labeled [$^{13}C_5$]-glutamine for 3 hours. Tumors from ketogenic-diet-fed mice exhibited approximately a 2-fold increase in glutamine uptake compared to controls, reflected in the levels of labeled and unlabeled glutamine (Figure S3A). Downstream metabolites including α-ketoglutarate and aspartate revealed greater M+3 enrichment in the former and M+2 and M+4 enrichment in the latter. These results indicate an accelerated rate of glutamine turnover in PDAC tumors under ketogenic conditions (Figures S3B and S3C).

To validate these findings *in vitro*, PDAC cells were cultured in media formulated to mimic ketogenic conditions. Compared to standard media, ketogenic-like media contain reduced glucose (2 mM) and elevated levels of fatty acids (2 mM), ketone bodies (5 mM), and glutamine (6 mM). Consistent with the *in vivo* results, cells cultured under ketogenic-like conditions displayed significantly higher ^{13}C-glutamine incorporation, reflected in an elevation in the M+5 isotopologue (Figure S4A). Further, there were increases in M+5, M+2, and M+3 labeling of relevant downstream TCA intermediates, reflecting enhanced glutamine utilization and turnover under ketogenic stress (Figures S4B–S4F).

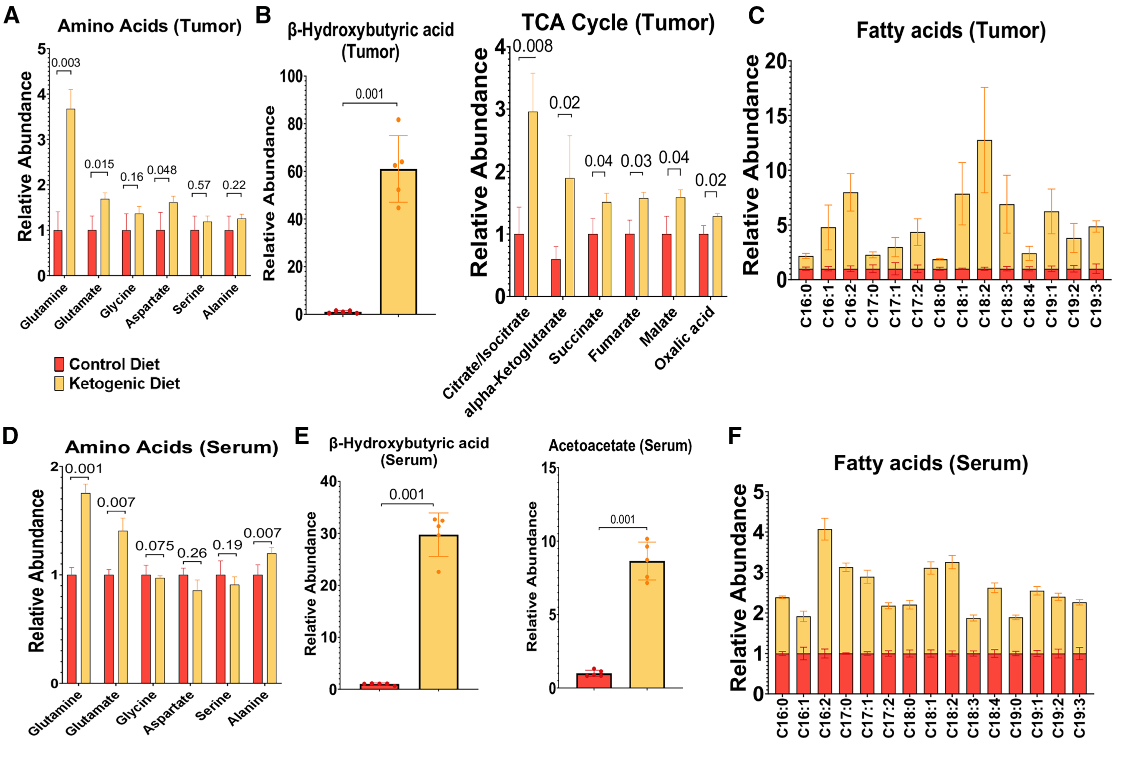

Figure 2. Impact of a ketogenic diet on TCA cycle and related metabolites in a PDAC mouse model

Metabolic profiling of subcutaneous MIA-PaCa2 tumor and serum samples from mice maintained on a ketogenic versus control diet (n = 5).

(A) Relative abundances of glutamine, glutamic acid, glycine, aspartate, serine, and alanine in tumor tissue.

(B) Levels of β-hydroxybutyric acid and TCA cycle intermediates in tumors.

(C) Comparison of tumor-associated fatty acid levels between ketogenic- and control-fed mice.

(D) Serum concentrations of amino acids.

(E) Circulating levels of ketone bodies in serum samples.

(F) Comparison of serum fatty acid profiles between mice on a ketogenic diet and those on a control diet.

As a control experiment to ensure that glutamine utilization was not merely a function of higher levels in the ketogenic-like media, compared to the control media, we repeated the experiment adjusting the glutamine levels in ketogenic-like media to 4 mM while preserving other key ketogenic features—elevated fatty acids (2 mM), ketone bodies (5 mM), and reduced glucose (2 mM). Isotope tracing was performed at 30 min, 1 h, 2 h, and 3 h to quantify the rate of ^{13}C-glutamine utilization, calculated as $\textit{Rate of } ^{13}C \textit{ uptake} = \frac{\Delta 13_C}{\Delta T(t_2 - t_1)}$. The M+0 glutamine fraction declined faster in ketogenic medium compared with standard DMEM (1.26 vs. 1.00 *fraction/h*), indicating an ~30% increase in overall glutamine utilization, corroborating ^{13}C labeling data at higher glutamine concentrations (Figure 4A). The enrichment rates of TCA cycle intermediates, as well as anaplerotic and downstream cataplerotic amino acid intermediates, were consistently higher under ketogenic conditions. For instance, a ~30% increase was observed in the enrichment of M+3 glutamate, most apparent by 3 h (Figure 4B). The production rate of M+3 α-ketoglutarate was approximately 25% faster in ketogenic-like media at 3 h compared with control cells (0.33 vs. 0.26 *fraction/h*) (Figure 4C). A similar rise was observed with M+2 and M+4 aspartate (0.035 vs. 0.01 *fraction/h*) (Figure 4D). Note, M+5 α-ketoglutarate and glutamate did not show higher levels of ^{13}C isotopes, most likely due to the rapid turnover of [^{13}C]-glutamine through the TCA cycle, which limits detection of early isotopologue formation. The absence of a difference in M+5 glutamine (Figure 4A) may be expected since this was the isotopologue delivered in bulk in the media for both groups.

To further assess the functional impact of these metabolite changes through the TCA cycle, we performed Seahorse assays to measure mitochondrial respiration and glycolytic activity. Cells cultured in ketogenic-like media exhibited increased oxygen consumption rate (OCR), indicating enhanced mitochondrial activity, and decreased extracellular acidification rate (ECAR), reflecting reduced glycolysis (Figures S4G and S4H).

Together, these findings indicate that ketogenic conditions enhance glutamine flux through the TCA cycle and downstream-related pathways, highlighting increased TCA utilization even under nutrient-matched conditions.

Specific nutrient components of a ketogenic diet alter the metabolism of PDAC cells

To investigate how PDAC cells metabolize key nutrients under ketogenic conditions, we performed a series of isotope tracing experiments using uniformly labeled glucose, caprylic acid, β-hydroxybutyrate (BHB), and glutamine as the key components or related metabolites in a ketogenic diet. Mechanistically,

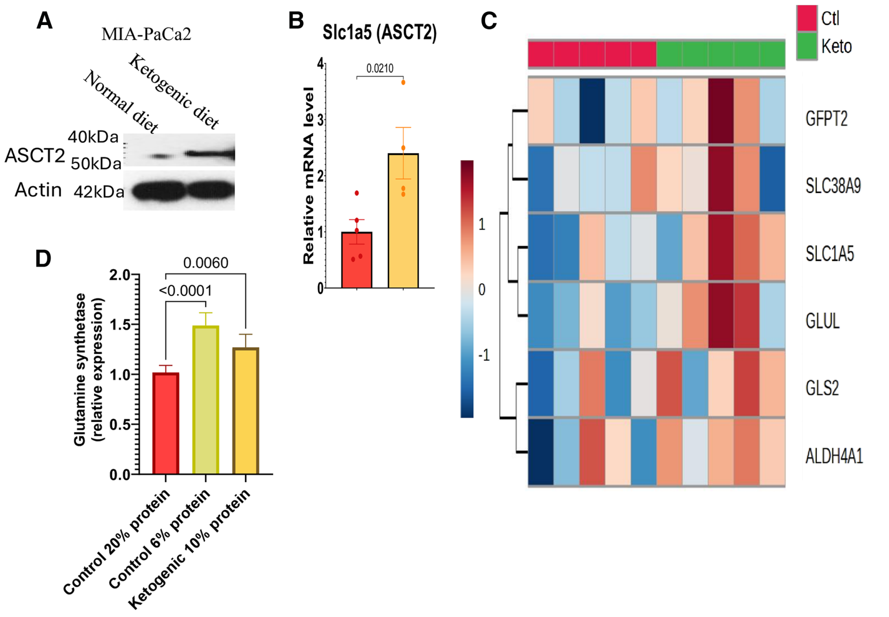

Figure 3. Liver-driven glutamine synthesis under protein restriction drives tumor glutamine utilization

(A and B) MIA-PaCa2 tumors were harvested, and levels of ASCT2 were measured by immunoblotting.

(C) RNA sequencing was conducted to determine glutamine utilizing pathway genes ($n = 5$).

(D) Levels of glutamine synthetase were measured in livers of mice exposed to diets that differed in protein content ($n = 5$).

glucose, caprylic acid, and BHB enter via acetyl-CoA, while glutamine is metabolized through glutamate to enter the TCA cycle at the level of α-ketoglutarate (Figure 5A). Tracing results revealed that glucose is the primary substrate for glycolytic intermediates, as shown by M+3 labeling of pyruvic acid and lactate from labeled glucose, while minimal labeling was observed from the other labeled substrates (Figures 5B and 5C). In contrast, caprylic acid, BHB, and glutamine contributed directly to the TCA cycle. This was apparent in the M+2-labeled metabolite levels from caprylic acid and BHB and M+5-labeled metabolites from glutamine (or M+4 citric acid) (Figures 5D–5F). As shown above for Figure 4, PDAC cells robustly imported glutamine into the TCA cycle, first converting the metabolite to glutamate (M+5) (Figure 5G) and then to downstream metabolites as M+4/5 isotopologues (Figures 5D–5F). We observed more than expected levels of M+5 citric acid, which suggests there is some reverse TCA flux from α-ketoglutarate to citric acid via reductive carboxylation (Figure 5D).

To assess cataplerosis (the exit of carbon from the TCA cycle), we focused on the M+2 and selected M+3 isotopologue patterns following administration of ketogenic components. Notably, carbons from BHB and caprylic acid were incorporated into cataplerotic metabolites glutamate and aspartate (Figures 5G and 5H). The contribution of [U-$^{13}C_5$]-glutamine to amino acid synthesis revealed it to be an important carbon source for glutamate, aspartate, and asparagine (Figures 5G–5I). Specifically, labeled glutamine contributed to aspartate (M+4) and asparagine (M+4) isotopologues.

Collectively, these data show that MIA PaCa-2 cells adapt to ketogenic stress by shifting from glycolysis to robust mitochondrial metabolism. PDAC cells efficiently metabolize ketone bodies, fatty acids, and especially glutamine to sustain TCA cycle activity. Importantly, elevated glutamine levels in tumors are not exclusively tumor-derived but also stem from systemic sources such as the liver.

A ketogenic diet potentiates glutamine metabolism inhibition of PDAC in mice

Based on these data, we reasoned that PDAC cells become reliant on glutamine in the context of a ketogenic diet and may be vulnerable to glutamine inhibition in combination with the diet. To test this possibility, we performed independent flank xenograft studies using human MIA-PaCa2 cells in nude mice and KPC cells in syngeneic C57BL/6 mice. In the former experiment, the pan-glutamine metabolism inhibitor DON was used, while a GLS inhibitor was used in the latter experiment. After 10 days of co-treatment, we observed a significant suppression of tumor growth in the combination therapy groups compared with a ketogenic diet or glutamine inhibition alone (Figures 6A and 6B). Measurements of β-hydroxybutyrate confirmed the expected metabolic effects of a ketogenic diet (Figure 6C). Body weights were slightly lower in mice receiving a ketogenic diet plus DON but were overall stable, indicating the diet plus drug combinations were well tolerated (Figure 6D). At the end of the study, toxicity studies included serum laboratory tests to assess liver, kidney, and overall physiological function. The results revealed no signs of toxicity from either monotherapy or combination treatment (Figures S5A and S5B). Thus, these findings support a model where limited glucose availability associated with a ketogenic diet shifts PDAC metabolism to rely on glutamine anaplerosis. This metabolic rewiring sensitizes PDAC tumors to glutamine metabolism inhibition.

DISCUSSION

KRAS-driven PDAC tumors (90% of all PDAC) rely on glutamine metabolism.[5,29–31] This finding is biologically relevant since glucose levels are already markedly depleted in the PDAC TME.[5,32] Studies reveal that KRAS-mutant PDAC tumors upregulate both GOT1 and GOT2 (glutamic acid oxaloacetate transaminase) to utilize glutamine to sustain the TCA cycle.[30] Thus, PDAC may be particularly susceptible to glutamine-metabolism-targeted treatments. Unfortunately, preclinical studies investigating glutamine inhibition as a therapeutic strategy have been equivocal. For instance, the oral glutaminase inhibitor CB-839 had no significant effect on tumor progression in LSL-KRAS^{G12D}; p53$^{L/+}$; PDX1-Cre genetically engineered mouse models or in human PDAC flank xenografts implanted in nude

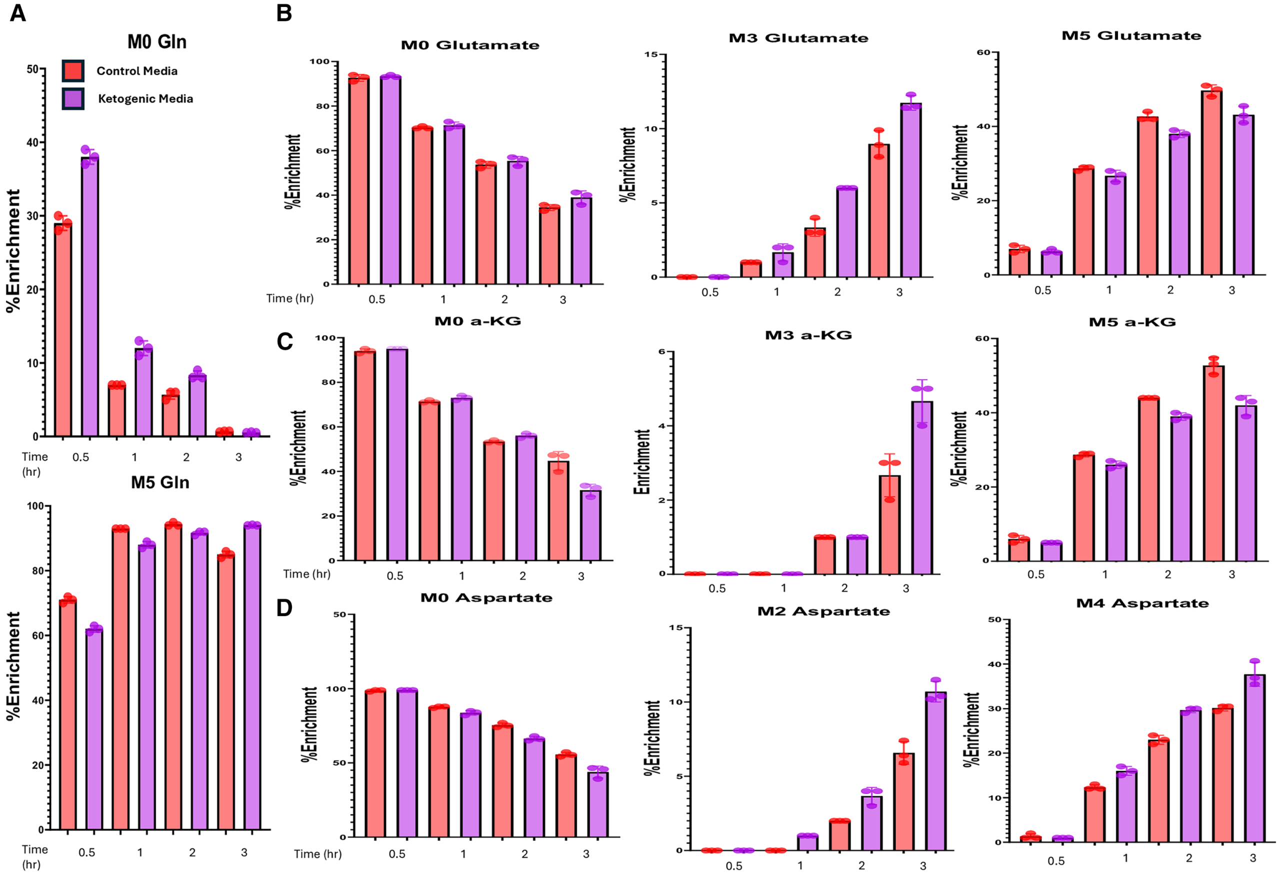

Figure 4. Ketogenic-like media enhances glutamine utilization in PDAC cells

Isotopologue enrichment over time is presented (n = 3).

(A) M+0 and M+5 glutamine input at 4 mM under control and ketogenic conditions.

(B) M+0, M+3, and M+5 isotopologues of glutamic acid.

(C) M+0, M+3, and M+5 isotopologues of α-ketoglutarate.

(D) M+0, M+2, and M+4 isotopologues of aspartate.

mice.[33] However, genetic targeting of the enzymes GOT1 and GOT2, which are downstream of glutamine metabolism, resulted in a significant decrease in tumor volume in a human PDAC xenograft model, indicating that these enzymes could serve as potential therapeutic targets.[30] Along these lines, treatment with the pan-glutamine-targeting drug DON resulted in a significant decrease in tumor volume across several PDAC orthotopic models.[34] Concerns regarding DON-related toxicity prompted the development of DRP-104, a pro-drug,[34] which showed promising responses in a syngeneic PDAC model.[35] Combination therapy with trametinib, an MEK inhibitor, significantly increased survival in the same model, indicating the superior potential of glutamine inhibition when combined with rationally selected complementary therapies.[35]

The success of glutamine metabolism inhibition in some preclinical studies spurred the pursuit of commercial development and testing in human clinical trials. As early as 1990, the potential of DON, a pan-glutamine metabolism inhibitor, was explored in late-stage clinical trials in sarcoma and mesothelioma patients. DON demonstrated poor efficacy, with one study of 36 evaluable sarcoma or mesothelioma cases reporting no objective response.[36] To mitigate the toxicity associated with glutamine inhibition, more selective enzyme-targeting drugs, such as GLS inhibitors, were developed. Telaglenastat (CB-839) has been the most widely studied glutaminase inhibitor in patients, with testing in 20 cancer-related clinical trials. Of these, 17 focused on solid tumors, and findings from four trials having been published to date. A phase 1 clinical trial evaluated CB-839 in addition to standard-of-care paclitaxel in patients with triple-negative breast cancer. Some patients experienced partial responses despite having paclitaxel refractory disease.[37] A phase 1–2 clinical trial utilizing CB-839 plus capecitabine demonstrated no efficacy by RECIST criteria in solid tumors, including the majority of colorectal, cholangiocarcinoma, breast, and gallbladder cancers. A subgroup analysis of PIK3CA-mutant colorectal cancer patients revealed a non-significant trend toward prolonged progression-free survival (24.8 vs. 16 weeks, p = 0.198).[37,38] Two published late-phase clinical trials evaluating telaglenastat in combination with other therapies in metastatic renal cell carcinoma provide

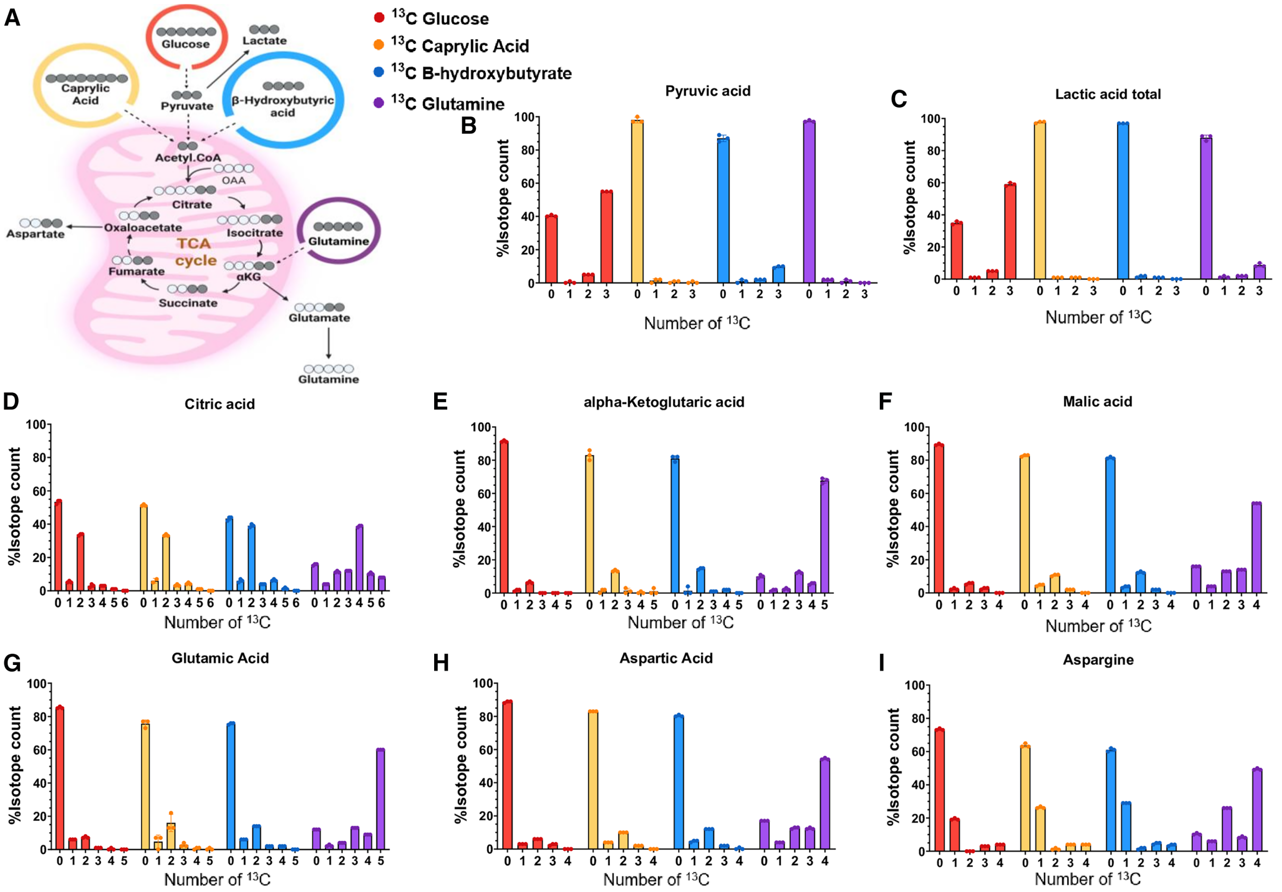

Figure 5. Contribution of ketogenic diet components to glycolysis, TCA cycle, and amino acid intermediates

The isotope counts of ^{13}C-caprylic acid, ^{13}C-glutamine, ^{13}C-glucose, and ^{13}C-β-hydroxybutyric acid were quantified using mass spectrometry in MIA-PaCa2 cells cultured with 4 mM [U-^{13}C] glutamine, 10 mM [U-^{13}C] DL-BHB, 2 mM [U-^{13}C] caprylic acid, and 5 mM [U-^{13}C] glucose for 3 h (*n* = 3 individual biological replicates).

(A) Schematic illustration of isotope tracing experiment.

(B–I) Pyruvic acid, lactic acid, citric acid, α-ketoglutaric acid, malic acid, glutamic acid, aspartic acid, and asparagine.

contradictory results. The phase 2 ENTRATA trial randomized 69 patients to telaglenastat plus everolimus vs. placebo plus everolimus. Everolimus is an mTORC1 inhibitor that disrupts glucose metabolism in cancer cells by reducing glucose uptake, perhaps sensitizing cancer cells to glutamine inhibition through similar mechanisms described in the present study.[39] Patients receiving telaglenastat plus everolimus had a small but non-significant improvement in progression-free survival (3.9 months vs. 1.9 months, p = 0.079).[40] The phase 3 CANTANA clinical trial evaluated telaglenastat plus cabozantinib vs. placebo plus cabozantinib in 444 patients with metastatic renal cell carcinoma. Cabozantinib is an MET and vascular endothelial growth factor receptor (VEGFR) inhibitor and has shown similar metabolic effects to everolimus in cancer cells, namely decreasing glucose utilization[41] accounting for the rationale for the combination. There was no difference in the primary endpoint of progression-free survival (p = 0.65).[42] In summary, telaglenastat seems to have marginal or no activity against cancer in patient studies, and optimal therapeutic combinations have not yet been identified.

Herein, we show that a ketogenic diet enhances glutamine metabolism in PDAC and leverage this finding into a therapeutic combination strategy. In the tested pre-clinical models, DON and CB-839 were both more effective in the context of a ketogenic diet. Findings were supported by metabolic studies in mice fed a ketogenic diet as well as *in vitro* isotope tracer studies using a customized media designed to reflect the key metabolic features of a ketogenic diet. It is important to note that while this study employed a medium-chain saturated fatty acid (caprylic acid), a ketogenic diet in patients is likely to elevate circulating levels of numerous different fatty acids derived from diverse dietary fat sources. Thus, the model here was a simplified simulation of the associated nutrient implications of a ketogenic diet.

Future studies could assess the impact of adding chemotherapy to this therapeutic strategy, since chemotherapy is known to further increase dependence on mitochondrial metabolism,[43] offering a multi-agent approach to optimize therapy (ketogenic diet + glutamine inhibition + chemotherapy). While chemotherapy and CB-839 have not shown substantial efficacy in combination to this point,[44,45] adding a ketogenic diet may

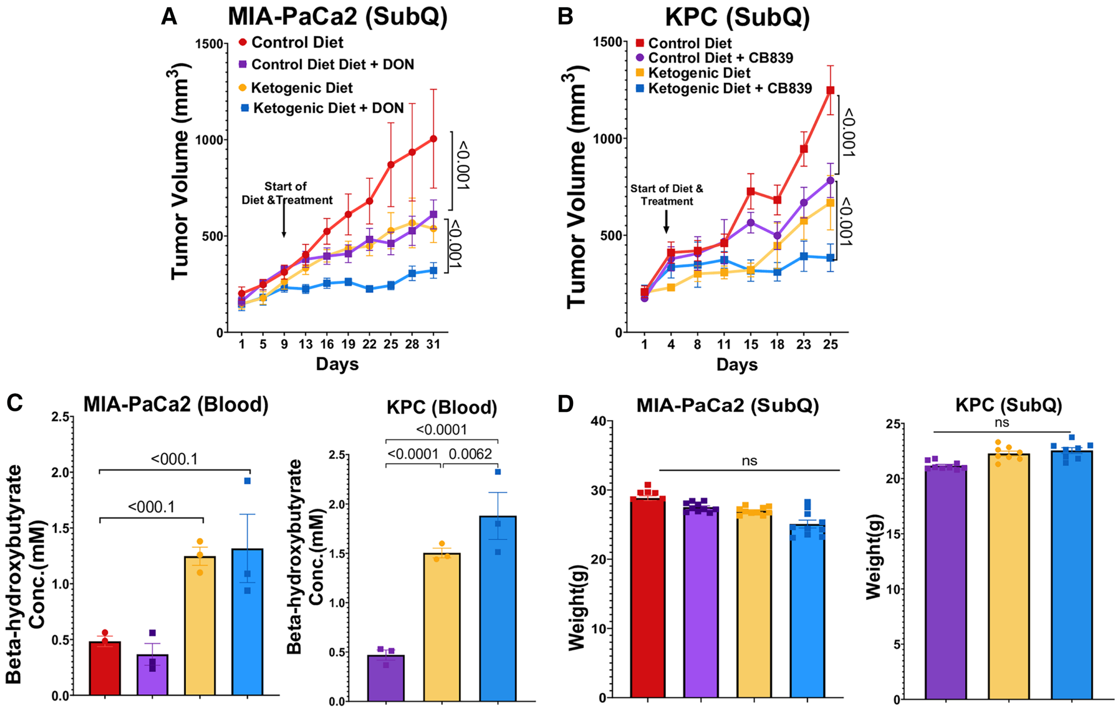

Figure 6. Synergistic inhibition of tumor growth with ketogenic diet and glutamine metabolism inhibitors

(A) MIA-PaCa2 tumor volumes in nude athymic mice treated with DON in combination with a ketogenic diet (*n* = 7).

(B) Subcutaneous KPC tumor volumes in C57BL/6 mice treated with CB-839 in combination with a ketogenic diet (*n* = 7).

(C and D) Measured levels of β-hydroxybutyrate and body weight (*n* = 7).

provide the necessary shift in metabolism to sensitize cancer cells to a meaningful degree. While our study primarily focused on the metabolic interplay between the ketogenic diet and glutamine metabolism, it is important to recognize that DON, as a glutamine analog, likely exerts its antiproliferative effects through broader mechanisms beyond glutaminase inhibition. One key pathway influenced by DON is *de novo* nucleotide biosynthesis, which is highly dependent on glutamine-derived nitrogen. In the context of a ketogenic diet—characterized by reduced glucose and protein intake—it is plausible that nucleotide synthesis also becomes further impaired. This compounded metabolic stress may enhance the anti-tumor efficacy of DON by disrupting both carbon and nitrogen sources essential for tumor growth. Future studies will be necessary to directly assess how ketogenic dietary stress alters nucleotide metabolism and to what extent suppression of nucleotide synthesis contributes to the therapeutic outcome. It will also be important to evaluate other metabolic modulators in combination with a ketogenic diet, including alternative drugs that target glutamine metabolism (JHU083, azaserine, and acivicin) or other enzymes fundamental to core metabolic pathways.[21]

Limitations of the study

Despite enhanced therapeutic efficacy, the combination of a ketogenic diet and glutamine metabolism inhibition did not eradicate tumors. The incomplete response suggests that PDAC metabolism can adapt by consuming other nutrients. Future experiments performed in the context of glutamine metabolism inhibition may uncover additional resistance pathways and metabolic vulnerabilities, further informing a multi-metabolic modulation approach. Additionally, implementing a ketogenic diet can be challenging for patients.[46] It remains unknown if the metabolic changes and safety profile observed here are generalized to patients receiving glutaminase inhibitors like CB-839 (telaglenastat). Newer agents like the prodrug, DRP-104, hold promise because of potential improvements in associated toxicities. Careful dose optimization in phase 1 studies of patients consuming a ketogenic diet will be important to consider. Further, a ketogenic diet may influence the TME beyond tumor intrinsic effects. Future investigations will aim to characterize how a ketogenic diet, both alone and in combination with glutamine-targeting therapies, modulates the immune composition (e.g., T cell and myeloid cell infiltration) in tumors, angiogenesis, and stromal signaling. Finally, in the isotope tracing experiment, caprylic acid (C8:0), a medium-chain fatty acid, was used as a representative fatty acid in the ketogenic media. Medium-chain fatty acids such as caprylic acid differ from long-chain fatty acids in their mitochondrial transport and metabolism; they enter mitochondria independently of the carnitine palmitoyl transferase (CPT) transport system, whereas long-chain fatty acids require CPT-mediated transport.[47,48] This property allows caprylic acid to undergo rapid β-oxidation and directly contribute acetyl-CoA to the TCA cycle. While generalizability of the simplified media model is uncertain, experiments repeated with long-chain fatty acids yielded similar metabolic changes.

CellPress
OPEN ACCESS

RESOURCE AVAILABILITY

Lead contact

Further information and request for resource and reagents should be directed to and will be fulfilled by the lead contact, Professor Jordan M. Winter (jordan.winter@uhhospitals.org).

Materials availability

This study did not generate new unique reagents.

Data and code availability

Metabolomics data have been deposited in the Metabolomics Workbench repository under the study identifiers ST004642 and ST004641. Tumor RNA sequencing data have been deposited in the NCBI Gene Expression Omnibus (GEO) under accession number GSE320214. This study did not generate custom code. Any additional information required to reanalyze the data reported in this study is available from the lead contact upon request.

ACKNOWLEDGMENTS

Grant support comes from the American Cancer Society MRSG-14-019-01-CDD, American Cancer Society 134170-MBG-19-174-01-MBG, Gateway for Cancer Research G-22-1100, NCI R37CA227865-01A1, NCI R01 CA281219, the Case Comprehensive Cancer Center GI SPORE 5P50CA150964-08, Case Comprehensive Cancer Center core grant P30CA043703, and University Hospitals research start-up package (J.M.W.). We are grateful for additional support from numerous donors to the University Hospitals Surgical Oncology Lab, including the John and Peggy Garson Family Research Fund; The Jerome A. and Joy Weinberger Family Research Fund; the Hieronymous Family; Robin Holmes-Novak, in memory of Eugene, Brittan, and Fred DiSanto; and Rosi and Saby Behar. Additional grant support for J.R.B. is provided by NIH-NCI R01 CA212600; U01CA224012-03; NIH-NCI R21 CA263996; the 2015 Pancreatic Cancer Action Network-AACR Research Acceleration Network Grant (grant number 15-90-25-BROD; the Lustgarten Foundation; and the Hirshberg Foundation.

AUTHOR CONTRIBUTIONS

Conceptualization, O.H. and J.M.W.; investigation, O.H., A.R., J.A.B., and M. MacArthur.; helped with experiments & validation, R.C., S.T., P.G., W.B., D.S., J.C., N.M., S.L., C.K., M.R., I.S., K.L., H.C., A.M., S.A., and K.J.; methodology, A.L., C.S.B., J.J.H., P.N., and C.H.; resources, R.W., A.V.-G., M. Miyagi, H.B., P.J.L., L.D.R., and J.D.R.; data curation, O.H., A.R., J.A.B., and M. MacArthur; writing – original draft, O.H. and J.M.W.; writing – review and editing, O.H., J.M.W., A.R., L.D.R., P.J.L., M.Z., and H.B.; funding acquisition, J.M.W.; supervision, O.H. and J.M.W.

DECLARATION OF INTERESTS

The authors declare no competing interests.

STAR★METHODS

Detailed methods are provided in the online version of this paper and include the following:

- KEY RESOURCES TABLE
- EXPERIMENTAL MODEL AND STUDY PARTICIPANTS DETAILS
  - Mice
  - Cell lines
- METHOD DETAILS
  - Pancreatic tumor models and endpoints
  - Cell culture treatments
  - Clonogenic assay
  - Drug administration in mouse studies
  - Infusion studies
  - Metabolite extraction and derivatization
  - Liquid chromatography-mass spectrometry (LC-MS)
  - Tumor RNA-Sequencing
- QUANTIFICATION AND STATISTICAL ANALYSIS
  - LC-MS data processing

SUPPLEMENTAL INFORMATION

Supplemental information can be found online at https://doi.org/10.1016/j.xcrm.2026.102770.

Received: October 31, 2024
Revised: November 25, 2025
Accepted: March 27, 2026
Published: April 23, 2026

REFERENCES

1. American Cancer Society (2023). Cancer Facts & Figures 2023 (American Cancer Society).
2. Ho, W.J., Jaffee, E.M., and Zheng, L. (2020). The tumour microenvironment in pancreatic cancer—clinical challenges and opportunities. Nat. Rev. Clin. Oncol. *17*, 527–540.
3. Zhang, T., Ren, Y., Yang, P., Wang, J., and Zhou, H. (2022). Cancer-associated fibroblasts in pancreatic ductal adenocarcinoma. Cell Death Dis. *13*, 897.
4. Vaziri-Gohar, A., Zarei, M., Brody, J.R., and Winter, J.M. (2018). Metabolic dependencies in pancreatic cancer. Front. Oncol. *8*, 617.
5. Kamphorst, J.J., Nofal, M., Commisso, C., Hackett, S.R., Lu, W., Grabocka, E., Vander Heiden, M.G., Miller, G., Drebin, J.A., Bar-Sagi, D., et al. (2015). Human pancreatic cancer tumors are nutrient poor and tumor cells actively scavenge extracellular protein. Cancer Res. *75*, 544–553.
6. Yang, J., Yang, J., Zhang, Q., Wang, J., Lou, Y., Hong, Z., Wei, S., Sun, K., Wang, J., Chen, Y., Sheng, J., et al. (2022). Dynamic profiling of immune microenvironment during pancreatic cancer development suggests early intervention and combination strategy of immunotherapy. J. Immunother. Cancer *10*, e004087.
7. Zarei, M., Hue, J.J., Hajihassani, O., Graor, H.J., Katayama, E.S., Loftus, A.W., Bajor, D., Rothermel, L.D., Vaziri-Gohar, A., and Winter, J.M. (2022). Clinical development of IDH1 inhibitors for cancer therapy. Cancer Treat Rev. *103*, 102334.
8. Weber, D.D., Aminzadeh-Gohari, S., Tulipan, J., Catalano, L., Feichtinger, R.G., and Kofler, B. (2020). Ketogenic diet in the treatment of cancer—Where do we stand? Mol. Metab. *33*, 102–121.
9. Lien, E.C., Westermark, A.M., Zhang, Y., Yuan, C., Li, Z., Lau, A.N., Sapp, K.M., Wolpin, B.M., and Vander Heiden, M.G. (2021). Low glycaemic diets alter lipid metabolism to influence tumour growth. Nature *599*, 302–307.
10. Hopkins, B.D., Pauli, C., Du, X., Wang, D.G., Li, X., Wu, D., Amadiume, S.C., Goncalves, M.D., Hodakoski, C., Lundquist, M.R., et al. (2018). Suppression of insulin feedback enhances the efficacy of PI3K inhibitors. Nature *560*, 499–503.
11. Yang, L., Teslaa, T., Ng, S., Nofal, M., Wang, L., Lan, T., Zeng, X., Cowan, A., McBride, M., Lu, W., et al. (2022). Ketogenic diet and chemotherapy combine to disrupt pancreatic cancer metabolism and growth. Med *3*, 119–136.e8.
12. Yang, H., Zingaro, V.A., Lincoff, J., Tom, H., Oikawa, S., Oses-Prieto, J.A., Edmondson, Q., Seiple, I., Shah, H., Kajimura, S., et al. (2024). Remodelling of the translatome controls diet and its impact on tumorigenesis. Nature *631*, 123–130.
13. Hui, S., Cowan, A.J., Zeng, X., Yang, L., TeSlaa, T., Li, X., Bartman, C., Zhang, Z., Jang, C., Wang, L., et al. (2020). Quantitative fluxomics of circulating metabolites. Cell Metab. *32*, 676–688.e4.
14. Mukherjee, P., Augur, Z.M., Li, M., Hill, C., Greenwood, B., Domin, M.A., Kondakci, G., Narain, N.R., Kiebish, M.A., Bronson, R.T., et al. (2019). Therapeutic benefit of combining calorie-restricted ketogenic diet and

glutamine targeting in late-stage experimental glioblastoma. Commun Biol *2*, 200. https://doi.org/10.1038/s42003-019-0455-x.

15. MacArthur, M.R., Mitchell, S.J., Chadaideh, K.S., Treviño-Villarreal, J.H., Jung, J., Kalafut, K.C., Reynolds, J.S., Mann, C.G., Trocha, K.M., Tao, M., et al. (2022). Multiomics assessment of dietary protein titration reveals altered hepatic glucose utilization. Cell Rep. *40*, 111187.
16. Encarnación-Rosado, J., Sohn, A.S., Biancur, D.E., Lin, E.Y., Osorio-Vasquez, V., Rodrick, T., Gonzalez-Baerga, D., Zhao, E., Yokoyama, Y., Simeone, D.M., et al. (2023). Targeting pancreatic cancer metabolic dependencies through glutamine antagonism. Nat. Cancer *4*, 104–120.
17. Biancur, D.E., Paulo, J.A., Małachowska, B., Quiles Del Rey, M., Sousa, C.M., Wang, X., Sohn, A.S.W., Chu, G.C., Gygi, S.P., Harper, J.W., et al. (2017). Compensatory metabolic networks in pancreatic cancers upon perturbation of glutamine metabolism. Nat. Commun. *8*, 15965.
18. Leone, R.D., Zhao, L., Englert, J.M., Sun, I.M., Oh, M.H., Sun, I.H., Arwood, M.L., Bettencourt, I.A., Patel, C.H., Wen, J., et al. (2019). Glutamine blockade induces divergent metabolic programs to overcome tumor immune evasion. Science *366*, 1013–1021.
19. Recouvreux, M.V., Grenier, S.F., Zhang, Y., Esparza, E., Lambies, G., Galapate, C.M., Maganti, S., Duong-Polk, K., Bhullar, D., Naeem, R., et al. (2023). Glutamine mimicry suppresses tumor progression through asparagine metabolism in pancreatic ductal adenocarcinoma. Nat. Cancer *4*, 234–247.
20. Zhao, Y., Butler, E.B., and Tan, M. (2019). Colorectal cancers utilize glutamine as an anaplerotic substrate of the TCA cycle in vivo. Sci. Rep. *9*, 19164.
21. Vaziri-Gohar, A., Cassel, J., Mohammed, F.S., Zarei, M., Hue, J.J., Hajihassani, O., Graor, H.J., Srikanth, Y.V.V., Karim, S.A., Abbas, A., et al. (2022). Limited nutrient availability in the tumor microenvironment renders pancreatic tumors sensitive to allosteric IDH1 inhibitors. Nat. Cancer *3*, 852–865.
22. Boison, D. (2017). New insights into the mechanisms of the ketogenic diet. Curr. Opin. Neurol. *30*, 187–192.
23. Oliveira, C.L.P., Mattingly, S., Schirrmacher, R., Sawyer, M.B., Fine, E.J., and Prado, C.M. (2018). A nutritional perspective of ketogenic diet in cancer: A narrative review. J. Acad. Nutr. Diet. *118*, 668–688.
24. Poff, A.M., Ward, N., Seyfried, T.N., Arnold, P., and D'Agostino, D.P. (2015). Non-toxic metabolic management of metastatic cancer in VM mice: Ketogenic diet, ketone supplementation, and hyperbaric oxygen therapy. PLoS One *10*, e0127407.
25. Allen, B.G., Bhatia, S.K., Anderson, C.M., Eichenberger-Gilmore, J.M., Sibenaller, Z.A., Mapuskar, K.A., Schoenfeld, J.D., Buatti, J.M., Spitz, D.R., and Fath, M.A. (2014). Ketogenic diets as an adjuvant cancer therapy: History and potential mechanism. Redox Biol. *2*, 963–970.
26. Scheck, A.C., Abdelwahab, M.G., Fenton, K.E., and Stafford, P. (2012). The ketogenic diet for the treatment of glioma. Epilepsy Res. *100*, 327–337.
27. Ferrer, M., Mourikis, N., Davidson, E.E., Kleeman, S.O., Zaccaria, M., Habel, J., Rubino, R., Gao, Q., Flint, T.R., Young, L., et al. (2023). Ketogenic diet promotes tumor ferroptosis but induces relative corticosterone deficiency that accelerates cachexia. Cell Metab. *35*, 1147–1162.e7.
28. Wang, Y., and Watford, M. (2007). Glutamine, insulin and glucocorticoids regulate glutamine synthetase expression. Biochim. Biophys. Acta *1770*, 594–600.
29. Jin, J., Byun, J.K., Choi, Y.K., and Park, K.G. (2023). Targeting glutamine metabolism as a therapeutic strategy for cancer. Exp. Mol. Med. *55*, 706–715.
30. Son, J., Lyssiotis, C.A., Ying, H., Wang, X., Hua, S., Ligorio, M., Perera, R.M., Ferrone, C.R., Mullarky, E., Shyh-Chang, N., et al. (2013). Glutamine supports pancreatic cancer growth through a KRAS-regulated metabolic pathway. Nature *496*, 101–105.
31. Wise, D.R., and Thompson, C.B. (2010). Glutamine addiction: A new therapeutic target in cancer. Trends Biochem. Sci. *35*, 427–433.
32. Vaziri-Gohar, A., Hue, J.J., Abbas, A., Graor, H.J., Hajihassani, O., Zarei, M., Titomihelakis, G., Feczko, J., Rathore, M., Chelstowska, S., et al. (2023). Increased glucose availability sensitizes pancreatic cancer to chemotherapy. Nat. Commun. *14*, 3823.
33. Earhart, R.H., Amato, D.J., Yuang-Chi Chang, A., Borden, E.C., Shiraki, M., Dowd, M.E., Comis, R.L., Davis, T.E., and Smith, T.J. (1990). Phase II trial of 6-diazo-5-oxo-L-norleucine versus aclacinomycin-A in advanced sarcomas and mesotheliomas. Invest. New Drugs *8*, 113–119.
34. DeMichele, A., Harding, J.J., Telli, M.L., Munster, P.N., McKay, R., Iliopoulos, O., Orford, K.W., Bennett, M.K., Mier, J.W., Owonikoko, T.K., et al. (2016). Phase 1 study of CB-839 with paclitaxel in triple-negative breast cancer. J. Clin. Oncol. *34*, 1011.
35. Zhao, Y., Feng, X., Chen, Y., Selfridge, J.E., Gorityala, S., Du, Z., Wang, J.M., Hao, Y., Cioffi, G., Conlon, R.A., et al. (2020). 5-fluorouracil enhances the antitumor activity of the glutaminase inhibitor CB-839. Cancer Res. *80*, 4815–4827.
36. Saxton, R.A., and Sabatini, D.M. (2017). mTOR signaling in growth, metabolism, and disease. Cell *168*, 960–976.
37. Lee, C.H., Motzer, R., Emamekhoo, H., Matrana, M., Percent, I., Hsieh, J.J., Hussain, A., Vaishampayan, U., Liu, S., McCune, S., et al. (2022). Telaglenastat plus everolimus in advanced renal cell carcinoma. Clin. Cancer Res. *28*, 3248–3255.
38. Emberley, E., Pan, A., Chen, J., Dang, R., Gross, M., Huang, T., Li, W., MacKinnon, A., Singh, D., Sotirovska, N., Steggerda, S.M., et al. (2021). Telaglenastat enhances antitumor activity of signal transduction inhibitors. PLoS One *16*, e0255758.
39. Tannir, N.M., Agarwal, N., Porta, C., Lawrence, N.J., Motzer, R., McGregor, B., Lee, R.J., Jain, R.K., Davis, N., Appleman, L.J., et al. (2022). Telaglenastat plus cabozantinib in renal cell carcinoma. JAMA Oncol. *8*, 1411–1418.
40. Zarei, M., Hajihassani, O., Hue, J.J., Graor, H.J., Loftus, A.W., Rathore, M., Vaziri-Gohar, A., Asara, J.M., Winter, J.M., and Rothermel, L.D. (2022). Wild-type IDH1 inhibition enhances chemotherapy response in melanoma. J. Exp. Clin. Cancer Res. *41*, 283.
41. Eads, J.R., Krishnamurthi, S.S., Saltzman, J.N., Bajor, D.L., Vinayak, S., Barnholtz-Sloan, J., Meropol, N.J., Markowitz, S.D., and Wang, Z. (2018). Phase I trial of CB-839 plus capecitabine in solid tumors. J. Clin. Oncol. *36*, 2562.
42. Zarei, M., Hajihassani, O., Hue, J.J., Loftus, A.W., Graor, H.J., Nakazzi, F., Naji, P., Boutros, C.S., Uppin, V., Vaziri-Gohar, A., et al. (2024). IDH1 inhibition potentiates chemotherapy efficacy in pancreatic cancer. Cancer Res. *84*, 3072–3085.
43. Khodabakhshi, A., Akbari, M.E., Mirzaei, H.R., Mehrad-Majd, H., Kalamian, M., and Davoodi, S.H. (2020). MCT-based ketogenic diet for breast cancer treatment. Nutr. Cancer *72*, 627–634.
44. Schönfeld, P., and Wojtczak, L. (2016). Short- and medium-chain fatty acids in energy metabolism. J. Lipid Res. *57*, 943–954.
45. Chen, J., Faller, D., and Spanjaard, R. (2005). Short-chain fatty acid inhibitors of histone deacetylases. Curr. Cancer Drug Targets *3*, 219–236.
46. Zarei, M., Hajihassani, O., Hue, J.J., Graor, H.J., Rothermel, L.D., and Winter, J.M. (2023). Targeting wild-type IDH1 enhances chemosensitivity in pancreatic cancer. Preprint at bioRxiv. https://doi.org/10.1101/2023.03.29.534596.
47. Hue, J.J., Graor, H.J., Zarei, M., Katayama, E.S., Ji, K., Hajihassani, O., Loftus, A.W., Vaziri-Gohar, A., and Winter, J.M. (2022). IDO1 is a therapeutic target for pancreatic cancer–associated depression. Mol. Cancer Ther. *21*, 1810–1822.
48. Rathore, M., Zhang, W., Wright, M., and Zarei, M. (2022). Liver endothelium microenvironment promotes HER3-mediated cell growth in pancreatic ductal adenocarcinoma. J. Cancer Sci. Clin. Ther. *06*.
49. Hue, J.J., Zarei, M., Graor, H.J., Hajihassani, O., Katayama, E.S., Loftus, A.W., Vaziri-Gohar, A., and Winter, J.M. (2022). Surgical techniques and tips for murine models of pancreatic cancer. Preprint at bioRxiv. https://doi.org/10.1101/2022.07.30.502136.

STAR★METHODS

KEY RESOURCES TABLE

REAGENT or RESOURCE	SOURCE	IDENTIFIER
**Chemicals, peptides, and recombinant proteins**		
6-diazo-5-oxo-L-norleucine (DON)	Sigmaaldrich	D2141-25mg
CB-839	Medchem	HY-12248
Caprylic Acid	Cambridge Isotope	CAS#23G-0425
(±)-Sodium 3- hydroxybutyrate	Cambridge Isotope	CAS# 2483735-72-2
D-Glucose	Cambridge Isotope	CAS# CLM-1396-0
L-Glutamine	Cambridge Isotope	CAS# CLM1822-H
ASCT2 (V501) Antibody #5345	Cell Signaling	RRID: AB_3741659
Ketogenic Diet	Bioserve	F366
Control Diet	Bioserve	F0761
**Critical commercial assays**		
Blood glucose/ketone monitoring system (ABBOTT)	Abbott	Ref. 98814-65
Blood glucose test strips (ABBOTT)	Abbott	Ref. 9972865
**Experimental models: Cell lines**		
MIA PaCa-2	ATCC	CRL-1420
KPC K8484	Gift	Darren Carpizo Laboratory
**Experimental models: Organisms/strains**		
Athymic nude mouse	Jackson Laboratory	CAT:002019
C57BL/6J mouse strain<br>*Mus musculus*, C57BL/6J	Jackson Laboratory	CAT#:000664
**Software and algorithms**		
El-Maven v0.12.0	N/A	https://github.com/ElucidataInc/ElMaven/releases
Thermo Xcalibur Qual Browser v4.5.474.0	Thermo Fisher Scientific	https://www.thermofisher.com
Metaboanalyst v5.0	N/A	https://www.metaboanalyst.ca/
Prism v10.4.1	Graphpad software	N/A
**Deposited data**		
Metabolomics Workbench	This paper	ST004642, ST004641
RNA sequencing	This paper	GEO: GSE320214

EXPERIMENTAL MODEL AND STUDY PARTICIPANTS DETAILS

Mice

All experiments involving mice were conducted under the approval of Case Western Reserve University Institutional Animal Care Regulations and Use Committee (CWRU; IACUC protocol no. 2018-0063 and Princeton University; IACUC protocol no. 3111). Six-to-eight-week-old, female, athymic nude mice (Foxn1 nu/nu) were purchased from Harlan Laboratories (no. 6903M) through ARC CWRU. Mice were sustained in the humidity-controlled animal facility with standard chow (Lab Diet, Prolab IsoPro RMH3000), ALPHA-dri bedding (nutrient-free), and under pathogen-free conditions. No additional nutrient-contained bedding or food was provided to the animals during these studies. Mice were fed *ad libitum*.

Cell lines

Reagents are listed in key resources table. The human pancreatic cancer cell line MIA PaCa-2 was obtained from the American Type Culture Collection (ATCC) (no. CRL-1420). The murine pancreatic cancer cell line (KPC K8484: Kras$^{G12D/+}$; Trp53$^{R172H/+}$; Pdx1-Cre) was provided by the Darren Carpizo and Eric Collisson laboratories. These two cell lines were maintained at conditions of 37°C and 5% CO_2. All cells were cultured in DMEM containing 4 mM glutamine and 25 mM glucose, supplemented with 1% penicillin/streptomycin, 10% FBS, and prophylactic doses of plasmocin (Life Technologies, no. MPP-01-03) to prevent mycoplasma infection. A MycoAlert detection kit (Lonza) was subsequently utilized for Mycoplasma screening.

METHOD DETAILS

Pancreatic tumor models and endpoints

For subcutaneous xenograft experiments, MIA PaCa-2 cells were suspended in 200 μL of a PBS: Matrigel solution (1:1). 1×10^6 suspended cells were injected subcutaneously into the right flank of mice. The KPC allograft tumor study was conducted in eight-week-old syngeneic female C57BL/6 mice.[49] 5×10^4 KPC cells suspended in Matrigel were injected subcutaneously in the right flank. In orthotopic syngeneic experiments, a suspension of 1:1 Matrigel with PBS and 5×10^4 KPC cells expressing Luciferase was injected directly into the mouse pancreas as previously described.[49] On the 10th day after the surgery, the presence of pancreatic tumors was confirmed using bioluminescence imaging (BLI) via Spectrum CT (PerkinElmer, 2898979) after injecting 100 μL D-luciferin (50 mg/mL in PBS) intraperitoneally. Tumor volumes were measured twice per week using a caliper (volume = length x width[1]/2); body weights were measured twice per week. Serum ketone and glucose levels were measured once per week via tail vein sampling using the Precision Xtra Glucose and Ketone Monitoring System from Abbott (no. 98814-65), as well as the Precision Xtra Blood Glucose and Ketone Test Strips from Abbott (no. 9972865; no. 7074565).

Cell culture treatments

To simulate low-glucose conditions in a pancreatic cancer microenvironment, glucose withdrawal (i.e., reduced glucose levels in the media) was performed as indicated. For low-glucose experiments, glucose-free DMEM (Life Technologies, no. 21013-024) was supplemented with 10% FBS and penicillin/streptomycin, and 2.5 mM glucose. The ketogenic-like cell culture medium consisted of 2 mM fatty acids, 2 mM glucose, 5 mM sodium β-hydroxybutyrate, and 6 mM glutamine. Generally, the fatty acid composition in the ketogenic-like media included a combination of short-, medium- and long-chain species, including butyric, heptanoic, caprylic, palmitic, oleic, and linoleic acids. In the isotope tracing study mapping the distribution of labeled carbon from fatty acids into downstream metabolites, the ketogenic media included caprylic acid only.

Clonogenic assay

Cells were plated in six-well plates at 1,500 cells per well. Cells were first cultured with media for 24 hours, followed by treatment under varying levels of glutamine, 10% FBS, and the indicated glucose concentrations. At the conclusion of experiments, colonies were fixed in a reagent containing 80% methanol and stained with 0.5% crystal violet. To determine relative growth, dye was dissolved from stained colonies with 10% acetic acid and the associated absorbance was measured using a microplate reader at 600 nm (GloMax Explorer system, Promega).[49]

Drug administration in mouse studies

In therapeutic experiments, treatments started once subcutaneous tumors were palpable, with tumor volumes averaging 120–150 mm^3 across treatment groups.[47,49] KPC tumors in C57BL/6J mice (n = 36) were randomized to the following treatment arms: control diet (n = 9), ketogenic diet (KD) (n = 10), control diet + CB-839 (n = 8), KD + CB-839 (n = 9). Treatments were administered after two initial tumor measurements to confirm consistent tumor growth. CB-839 was dissolved in a 3 mL solution consisting of 2 mL of vehicle (sterilized H_2O, NaCl, polyethylene glycol, Tween 80) and 1 mL of corn oil. Both the control and CB-839 (200 mg/kg) treatment group mice were treated via oral gavage three times per week. Experiments testing DON were performed in nude mice, with groups randomized to control diet (BioServ, no. F3197) + vehicle (sterilized H_2O, n = 5), ketogenic diet + vehicle (KD; BioServ, no. F3666, n = 5), control diet + DON (n = 8), and KD + DON (n = 9). After confirming the tumor progression by two initial tumor measurements to confirm tumor growth, treatments were administered. 2 mg of DON was suspended in 4 mL of vehicle for the dissolution of DON. Both vehicle (control) and DON treatments were administered interperitoneally (IP) twice per week at a dose of 5 mg/kg, unless indicated. Upon finalization of animal experiments, mice were euthanized using isoflurane inhalation followed by cervical dislocation. Tumor volume was assessed for each mouse and plotted longitudinally.

Infusion studies

To quantify the uptake of glutamine by PDAC tumors, 14-week-old female nude mice (Jax # 002019) bearing flank MIA PaCa-2 tumors were catheterized in the right jugular vein. After five days of recovery, mice were randomly assigned to receive either control (AIN93-M F3155, bioserv) or a ketogenic diet (S3666, bioserv). Mice were infused after 3 weeks on diets when tumors reached an average volume of 200 mm^3. On the day of the infusion, [U-^{13}C] Glutamine tracer (CLM-1822, Cambridge Isotope Laboratories) was prepared at 100mM in sterile saline. Mice were fasted around 1:30 p.m. and the infusion began at 3:30 p.m. at a minimally perturbative rate of 0.1 μl/min/g. Mice were infused for 2.5 h followed by tail blood collection, euthanasia by cervical dislocation and tissue harvesting. The contribution of glutamine to downstream metabolites is calculated by the metabolite labeling enrichment normalized to the tracer serum enrichment at 2.5 h. Cells were incubated with 2 mM caprylic acid (CIL CAS#23G-0425), 10 mM (±)-Sodium 3-hydroxybutyrate (CIL CAS# 2483735-72-2), 5mM glucose (CIL CAS# CLM-1396-0) and 4 mM of glutamine (CIL CAS# CLM1822-H), all with fully labeled carbons for 3 h.

Metabolite extraction and derivatization

Culture media was aspirated from each well of a six-well plate, and the cells were gently washed with 2 mL of saline solution. The plate was subsequently positioned carefully on ice. A lysis solution consisting of ice-cold methanol, ice-cold water, and 1 mM

tricarballylic acid in a 40:20:1 ratio (600 μL per well) was added. Cells were then delicately scraped using a cell scraper while maintaining the plate on ice. The cell suspension underwent vortexing for 10 s and centrifugation at 14,000 × g for 10 min at 4°C. The resulting supernatant was combined with 300 μL of chloroform, vigorously vortexed for 30 s, and centrifuged at 3,000 × g for 3 min at 4°C. The upper polar phase was collected and subjected to drying in a SpeedVac. Each sample underwent derivatization with 20 μL of 4% methoxyamine-hydrochloride in pyridine, followed by a 30 min incubation period at 45°C. Subsequently, the samples were further derivatized with 25 μL of mtBSTFA +1% t-BDMCS and incubated for 60 min at 45°C. After centrifugation at 14,000 × g for 10 min at 4°C, the supernatant was collected and processed for gas chromatography-mass spectrometry (GC-MS) analysis.

Liquid chromatography-mass spectrometry (LC-MS)

Metabolites were measured using GC-MS with an Agilent 5977B system, containing an HP-5 ms column (30 m × 0.25 mm, 0.25 μm). The injector temperature was maintained at 300°C, and 1 μL of each sample was injected. The GC temperature program started at 60°C, held for 1 min, increased by 6.5°C/min to 325°C, and maintained at 325°C for 10 min. Helium served as the carrier gas with a flow rate of 1.2 mL/min. The analytes underwent electron impact ionization (EI), and metabolite ions were monitored using selected ion monitoring (SIM) mode. The MS source and quadrupole temperature were set at 280°C and 150°C, respectively. The MassHunter software facilitated the annotation of metabolites, providing chromatographic peak areas for the monitored isotopomer peaks. Subsequently, IsoCorrectoR was employed to correct natural isotope abundance and derive the isotopomer distribution for each metabolite.

Tumor RNA-Sequencing

cDNA libraries were prepared from 100 ng enriched mRNA using the Ion Total RNA-Seq Kit v2. mRNA was fragmented, purified (Life Technologies Ambion), and adapter-ligated. Libraries were reverse transcribed and amplified using Ion Xpress Barcode primers and Platinum PCR SuperMix High Fidelity. Library yield and size distribution were assessed using an Agilent 2100 Bioanalyzer. Libraries with <50% of fragments in the 50–160 bp range proceeded to sequencing. Approximately 50 pM of pooled libraries were templated, enriched, and sequenced on an Ion S5 sequencer using the Ion 550 Chef kit and a configured Ion Torrent RNA-Seq run plan. Raw reads (fastq) were aligned to the mouse genome (mm10) using the Ion Torrent alignment tool. BAM files were analyzed using StrandNGS. Reads were filtered (alignment score ≥90, quality ≥10, ≤0 Ns, passed vendor QC) and normalized using DEseq. Differentially expressed genes (DEGs) between groups were identified using a Moderated t test ($p < 0.05$, fold change ≥2.0).

QUANTIFICATION AND STATISTICAL ANALYSIS

Data are presented as mean ± standard error of the mean (SEM) for all bar and line graphs. GraphPad Prism software (versions 9 and 10) was used for data visualization and statistical analysis. Sample sizes were determined based on pilot experiments and prior experience, with power calculations ($\alpha = 0.05$, power = 0.80) indicating that 5 mice per group were sufficient to detect biologically meaningful differences in tumor studies. Statistical comparisons between two groups were performed exclusively using two-tailed unpaired Student's t tests.

Survival analyses were carried out using Kaplan–Meier survival curves, with statistical significance assessed using the log rank (Mantel–Cox) test and adjusted for multiple comparisons using the Benjamini–Hochberg method where applicable. Pearson correlation coefficients were used to assess linear relationships between variables. Heatmaps and principal component analyses were generated using MetaboAnalyst version 5.0. All statistical tests were two-sided, and $p < 0.05$ was considered statistically significant.

LC-MS data processing

LC-MS analysis was used to measure water-soluble metabolites by running samples on the Orbitrap Exploris 480 mass spectrometer (Thermo Scientific) coupled with hydrophilic interaction chromatography (HILIC) and an XBridge BEH Amide column (150 mm × 2.1 mm, 2.5 μM particle size, Waters, Milford, MA). The gradient included solvent A (95%:5% $H2O$:acetonitrile with 20 mM ammonium acetate, 20 mM ammonium hydroxide, pH 9.4) and solvent B (100% acetonitrile) according to the following times and ratios: 0 min, 90% B; 2 min, 90% B; 3 min, 75% B; 7 min, 75% B; 8 min, 70% B, 9 min, 70% B; 10 min, 50% B; 12 min, 50% B; 13 min, 25% B; 14 min, 25% B; 16 min, 0.5% B, 20.5 min, 0.5% B; 21 min, 90% B; and 25 min, 90% B. The flow rate was 150 μL/min with an injection volume of 5 μL and a column temperature of 25°C. The MS scans were in polarity switching mode to enable both positive and negative ions across a mass range of 70–1000 m/z, with a resolution of 120,000. Data were analyzed using the El-MAVEN software (v 0.12.0, Elucidata).

Cell Reports Medicine, Volume *7*

Supplemental information

A ketogenic diet sensitizes pancreatic cancer to glutamine metabolism inhibitors

Omid Hajihassani, Asael Roichman, Jacob A. Boyer, Michal MacArthur, Ricardo Cordova, Alexander Loftus, Christina S. Boutros, Jonathan J. Hue, Parnian Naji, Soubhi Tahhan, Peter Gallagher, William Beegan, Danyal Shah, James Choi, Nimat Manzoor, Shihong Lei, Christine Kim, Moeez Rathore, Ishan Shah, Kevin Lebo, Helen Cheng, Anusha Mudigonda, Craig Hunter, Mehrdad Zarei, Sydney Alibeckoff, Karen Ji, Hallie Graor, Masaru Miyagi, Ali Vaziri-Gohar, Henri Brunengraber, Rui Wang, Peder J. Lund, Luke D. Rothermel, Joshua D. Rabinowitz, and Jordan M. Winter

Supplemental figure titles and legends:

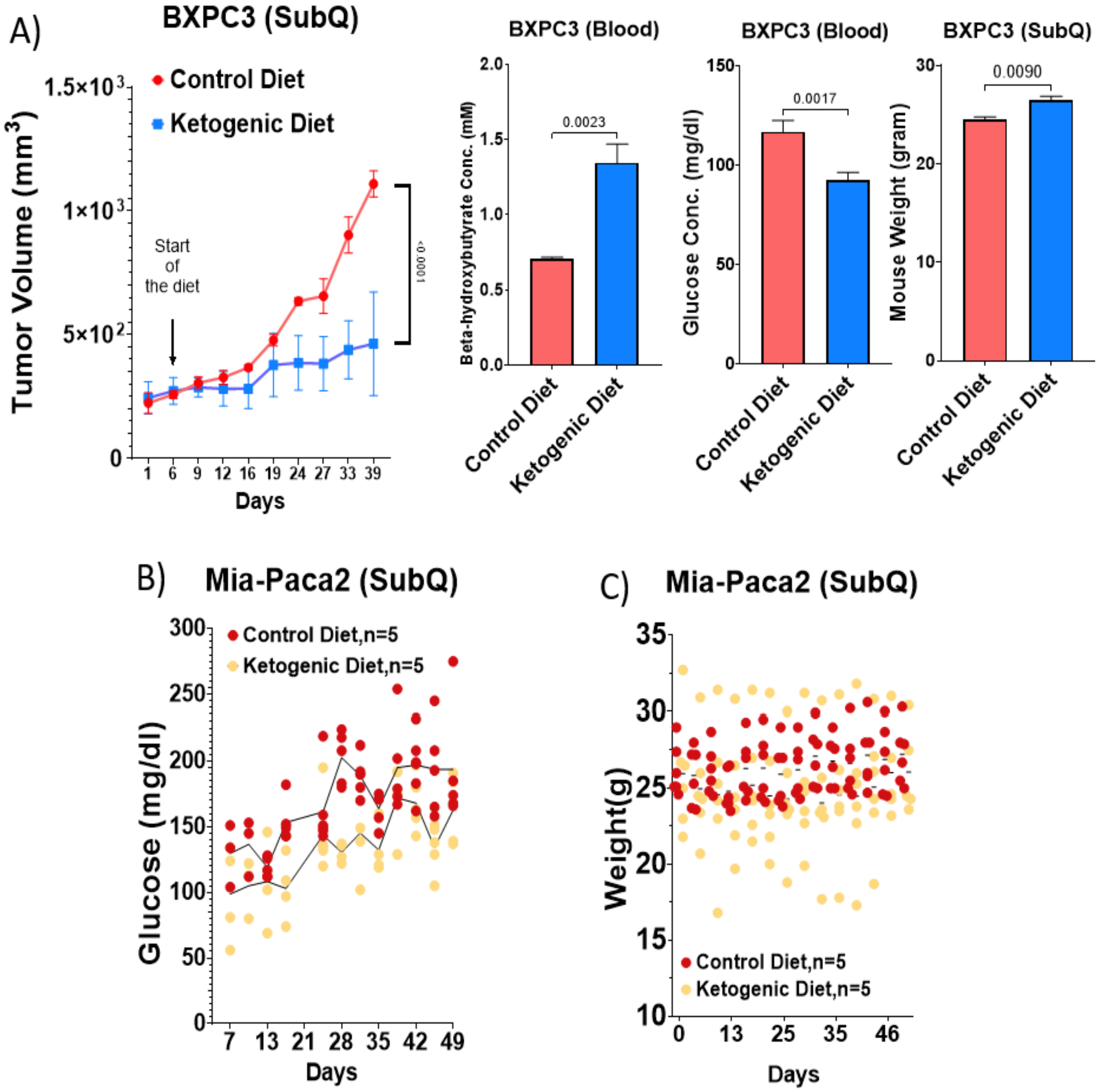

Supplementary Fig. 1: Mice maintained on a ketogenic diet reveal lower blood glucose levels while effectively managing their body weight. Related to figure 1. (A) BXPC3 tumors injected in the flank of athymic nude mice. Glucose levels were measured in individual mice throughout the experiment. During the experiment the levels of BHB, glucose and mouse weight were collected (n=5). **(B)** Tumor volumes per mouse are illustrated throughout the duration of the experiment, with each day's measurement representing n=5. **(C)** Mouse body weights were measured twice per week (n=5).

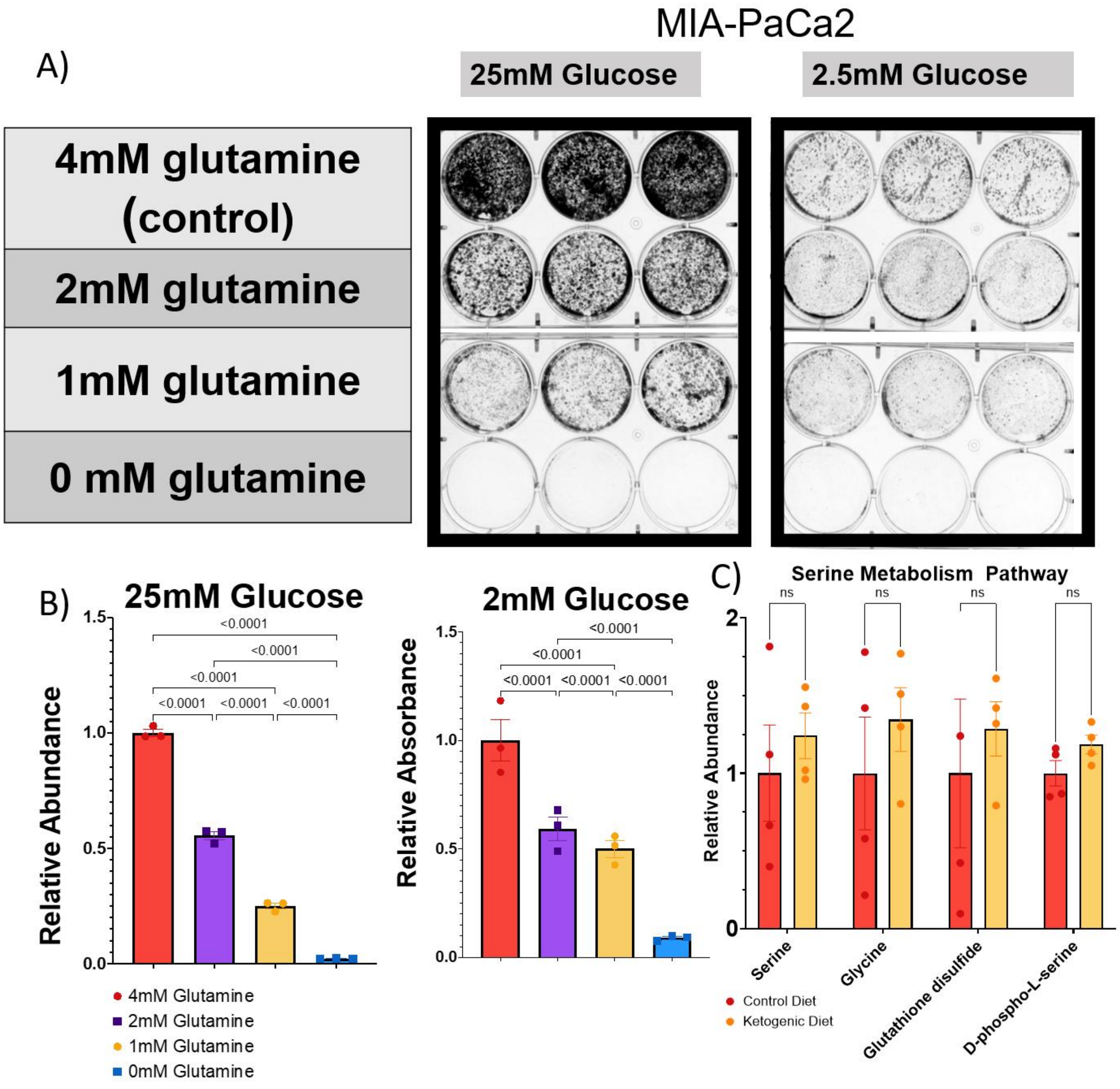

Supplementary Fig. 2: Glutamine is required for pancreatic cancer cells viability. Related to figure 2. Different glutamine levels were tested under high (25mM) and low (2.5mM) glucose conditions (n=3). **(B)** Quantitation of cell viability at the different indicated conditions (n=3). **(C)** Serine metabolism (n=3).

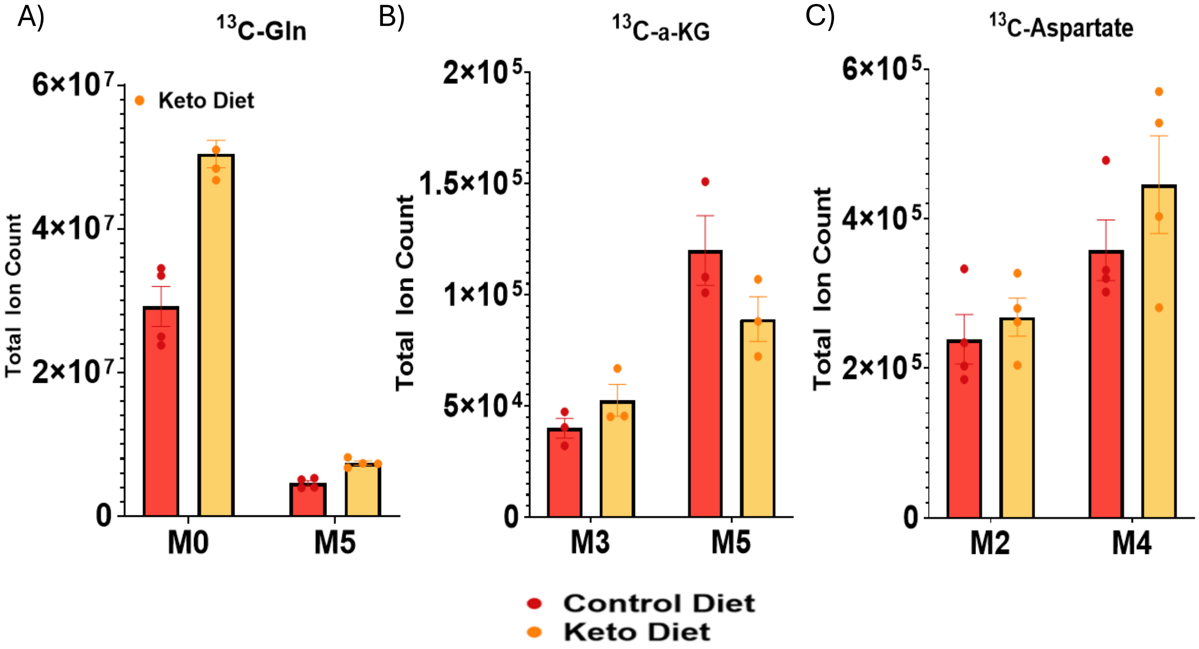

Supplementary Figure 3. PDAC tumors exhibit increase glutamine utilization under a ketogenic diet *in vivo.* Related to figure 4. Nude mice bearing PDAC flank tumors were injected with uniformly labeled [^{13}C]-glutamine and analyzed after 2.5 hr of tracer circulation(n=3). **(A)** M+0 and M+5 isotopologues of glutamine, and relevant isotopologues (n=3) of **(B)** α-ketoglutarate (n=3), and **(C)** aspartate (n=3).

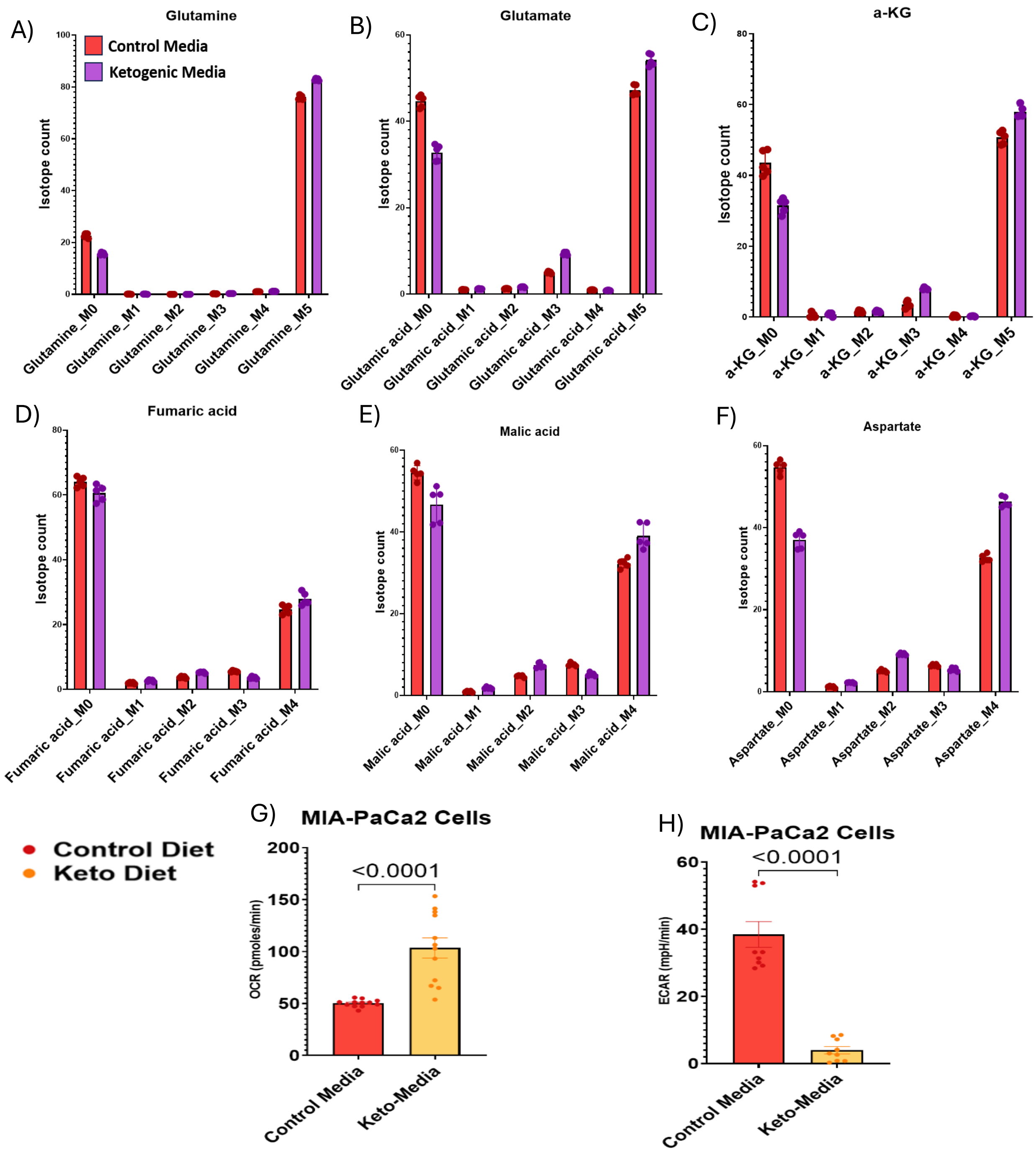

Supplementary Figure 4. PDAC cells maintain higher levels of TCA cycle metabolites under ketogenic conditions *in vitro.* Related to figure 5. Isotopologue analysis of [^{13}C]-glutamine metabolism was performed using 6 mM glutamine in ketogenic media and 4 mM glutamine in control media (n=3). **A)** M+0 through M+5 isotopologues of glutamine under control and ketogenic conditions (n=3). **B)** Glutamate, **C)** α-ketoglutarate, and M+0 through M+4 D**)** fumarate, **E)** malate, and **F)** aspartate. Seahorse quantified levels of **(G)** OCR **(H)** ECAR for cells treated with ketogenic media vs. cells treated with control media.

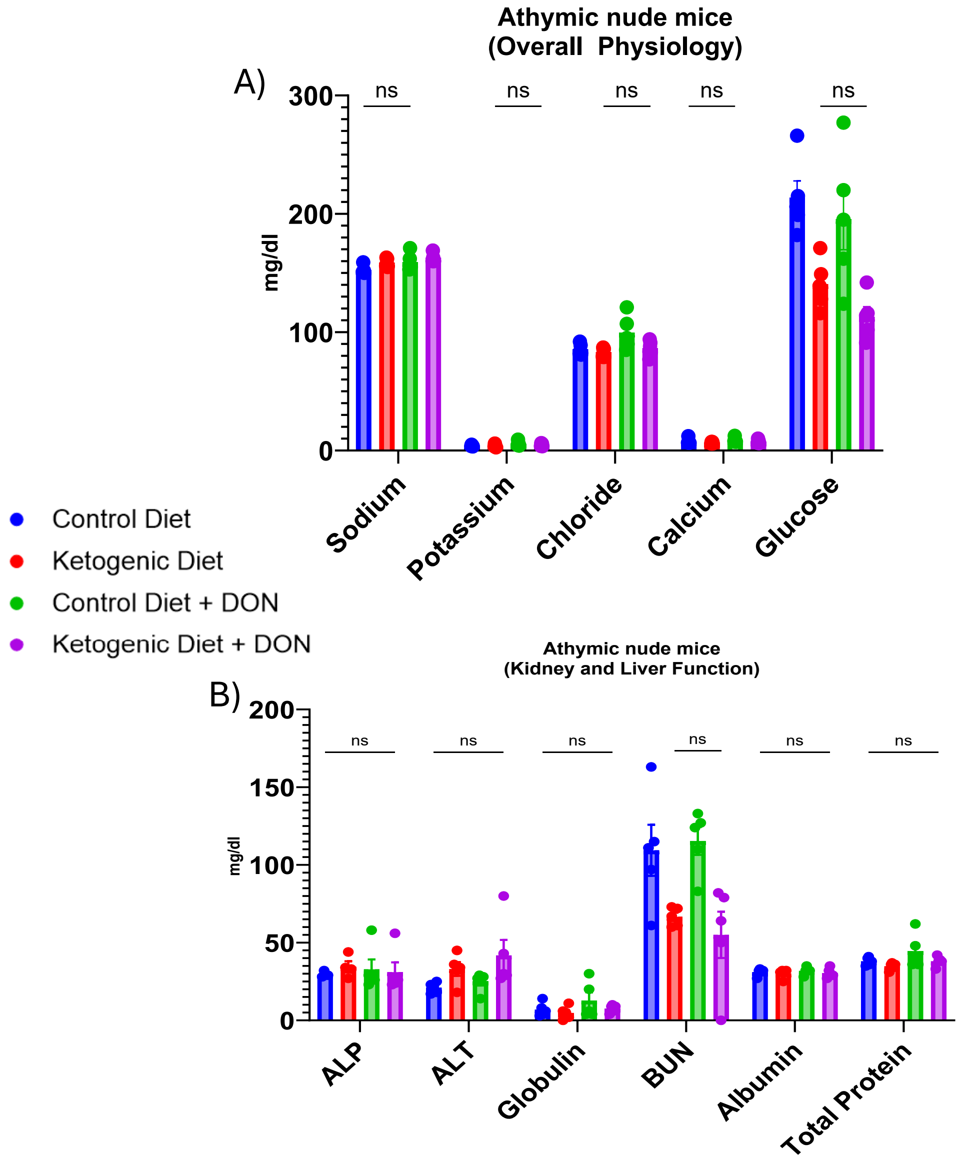

Supplementary Fig.5: Ketogenic diet and DON combination therapy reveals no significant toxicity. Related to figure 6. Tumor-bearing mice were evaluated (n=5). **(A)** Electrolyte and glucose levels reveal no significant differences in treatment groups. **(B)** Alkaline Phosphatase (ALP), Alanine aminotransferase (ALT), globulin, blood urea nitrogen (BUN), albumin, and total protein were measured as surrogate markers of liver and kidney function (n=5).
